# Supplementary material for: Assessing criticality in pre-seizure single-neuron activity of human epileptic cortex
Source: PLoS Comput Biol. 2021 Mar 8;17(3):e1008773. doi: 10.1371/journal.pcbi.1008773 (PMC7971851; doi:10.1371/journal.pcbi.1008773)
Supplement: S1 Text — (PDF) [file pcbi.1008773.s009.pdf]

# S1 Text

Based on the two exclusion criteria for MR estimation, a number of intracranial recordings were excluded:

**Contralateral hemisphere**: In total, out of 91 recordings from the entire MTL (16 reference, 75 pre-seizure), 4 had to be excluded because of criterion 1 and another 4 because of criterion 2, leaving a total of 83 recordings for which we could estimate the branching parameter $m$.

Splitting the recordings up into the different sub-regions of MTL results in a total number of recordings of $n = 212$ ($n_A = 68$, $n_H = 71$, $n_{EC} = 35$, $n_{PHC} = 38$). After applying the exclusion criteria, we obtained to $\tilde{n} = 184$ recordings, for which $m$ could be estimated ($\tilde{n}_A = 62$, $\tilde{n}_H = 56$, $\tilde{n}_{EC} = 32$, $\tilde{n}_{PHC} = 34$).

**Ipsilateral hemisphere**: In total, out of 105 recordings from the entire MTL (20 reference, 85 pre-seizure), 3 had to be excluded because of criterion 1 and another 9 because of criterion 2, leaving a total of 93 recordings for which we could estimate the branching parameter $m$.

Splitting the recordings up into the different sub-regions of MTL results in a total number of recordings of $n = 228$ ($n_A = 72$, $n_H = 84$, $n_{EC} = 34$, $n_{PHC} = 38$). After applying the exclusion criteria, we obtained to $\tilde{n} = 174$ recordings, for which $m$ could be estimated ($\tilde{n}_A = 51$, $\tilde{n}_H = 72$, $\tilde{n}_{EC} = 25$, $\tilde{n}_{PHC} = 26$).
