# Supplementary material for: Assessing criticality in pre-seizure single-neuron activity of human epileptic cortex
Source: PLoS Comput Biol. 2021 Mar 8;17(3):e1008773. doi: 10.1371/journal.pcbi.1008773 (PMC7971851; doi:10.1371/journal.pcbi.1008773)
Supplement: S1 Table — Recordings span both hemispheres (left and right) and different subregions of MTL, including hippocampus (H), amygdala (A), parahippocampal cortex (PHC) and entorhinal cortex (EC). Individual recordings of the same patient can span different subsets of the listed brain areas. Numbers of recordings after spike sorting, before applying exclusion criteria of MR estimation. The surgery outcomes were evaluated according to the Engel scale. No entry means that no surgery has been performed. Dataset provided by the Department of Epileptology in Bonn. (PDF) [file pcbi.1008773.s010.pdf]

patient ID	location of focus	reference recordings	pre-seizure recordings	brain regions	surgery outcome
1	L	1	3	LA, LPHC, RA, REC	1A
2	L	1	1	LH, LEC	-
3	L	1	3	LH, LPHC, RH, LA, RPHC, RA	3-4
4	L	1	4	LH, LEC, RH, RA, LA, LPHC, RPHC	-
5	L	1	2	LH, LPHC, RH, LA, RA	-
6	L	1	2	LA, LH, REC, RPHC, LEC	1A
7	R	1	1	LA, LH, RA, REC, RPHC	3
8	R	1	0	LA, LEC, RA, RH, REC	1A
9	L	1	3	LA, LH, LEC, RH, REC, RPHC	1A
10	R	1	3	LA, LH, LPHC, REC, RPHC, RH	1A
11	L	1	3	LA, RA, RH	1A
12	R	1	8	LA, LEC, LPHC, RA, RH, REC, RPHC, LH	1-2
13	R	1	5	LA, LH, LEC, LPHC, RA, RH, RPHC	1-2
14	L	1	3	LA, LH	1
15	R	1	6	LA, LH, LEC, LPHC, RA, RH, REC, RPHC	1-2
16	L	1	1	LH	1A
17	L	1	3	LH, RA, RH, REC, RPHC, LPHC, LA, LEC	1A
18	L	1	8	LA, LH, RA, LEC, RH, LPHC	1A
19	L	1	3	LA, LPHC	1A
20	L	1	25	LH, RA, RH, LA, LPHC	1-2
