# Supplementary material for: Assessing criticality in pre-seizure single-neuron activity of human epileptic cortex
Source: PLoS Comput Biol. 2021 Mar 8;17(3):e1008773. doi: 10.1371/journal.pcbi.1008773 (PMC7971851; doi:10.1371/journal.pcbi.1008773)
Supplement: Attachment — Submitted filename: 04202020_PLoSCB.pdf [file pcbi.1008773.s011.pdf]

The authors present a compact study which assesses the spike autocorrelation function during preictal and interictal periods and in different cortical regions. The study is motivated by the autocorrelation function's role in the context of critical transitions; the increasing timescale captured by the slower autocorrelation function decay when a critical transitions is approached is typically referred to as critical slowing down and may be used to infer the distance to criticality. Many epilepsy models assume a critical transition (or bifurcation) at seizure onset (see for example https://www.biorxiv.org/content/10.1101/2020.02.08.940072v1.full; also Jirsa et al., Brain, 2014) and the presence of critical slowing down prior to human seizures is currently a topic of debate and research. The present study touches on some of these timely questions by studying critical slowing down at the individual neuron dynamics near seizure onset. While the questions posed are interesting, the study unfortunately raises some methodological concerns and misses discussion of alternative explanations and key publications to put findings and their interpretation into a larger, more balanced context. The study may also benefit from a more comprehensive assessment that also includes ictal data and seizure offset. Nevertheless, the study is interesting and the use of individual neuron spiking and critical slowing down in epilepsy is novel. The authors should just extend their analyses to ictal data, be more cautious in their interpretations and provide a more balanced overview over the field (see comments below).

Main concerns:

1. Choice of methods:

The authors study the autocorrelation structure of single neuron spiking activity to probe proximity to a critical transition. As highlighted by Kramer et al., 2012 (also cited by the authors) a critical transition may be present even though single neurons do not capture critical slowing down. As further shown in Kramer et al., dynamical signatures such as critical slowing down may be better accessible in data like LFP, EEG. In the present paper the authors conclude that critical slowing down is not present solely based on the study of single neuron dynamics, which consequently poses a dilemma whether the conclusions by the authors (that there is no critical slowing down prior to seizure onset) are justified. In fact, several papers, which are currently not cited by the authors, seem to suggest otherwise, i.e. that LFP and EEG do indicate some changes potentially in line with critical slowing down:

[1] Chang, W.C., Kudlacek, J., Hlinka, J., Chvojka, J., Hadrava, M., Kumpost, V., Powell, A.D., Janca, R., Maturana, M.I., Karoly, P.J., Freestone, D.R., Cook, M.J., Palus, M., Otahal, J., Jefferys, J.G.R., Jiruska, P., 2018. Loss of neuronal network resilience precedes seizures and determines the ictogenic nature of interictal synaptic perturbations. Nat. Neurosci. 21 (12), 1742–1752 12.

[2] Meisel, C., Kuehn, C., 2012. Scaling effects and spatio-temporal multilevel dynamics in epileptic seizures. PLoS One 7, e30371.

[3] Maturana M. et al. Critical slowing down as a biomarker for seizure susceptibility. https://www.biorxiv.org/content/10.1101/689893v1 (paper is currently in print at Nat Comm 2020; DOI: 10.1038/s41467-020-15908-3).

I would suggest that, at the least, this dilemma, that single neuron activity may not be informative, as indicated by prior studies (e.g. Kramer et al., 2012), should be communicated transparently already in the introduction. Also, the authors should definitely provide a more balanced account on the literature citing also studies which may be more in line with indication of critical slowing down to balance their reporting. Currently, throughout the paper the authors predominantly only cite studies that did not find indication for critical slowing down at seizure onset, as for example:

…recent studies on seizure initiation did not find consistent warning signals of a critical transition prior to seizures [31,32]

2. Choice to time window:

The authors limit their investigation to a few minutes before seizure onset and leave out the seconds right before seizure onset (due to understandable methodological constraints). However, it should be made more clear that this approach therefore limits the analysis to a particular

(somewhat arbitrary) time window; if critical slowing down occurred at longer or shorter timescales, it would be missed by the present analysis. This is particularly important since recent comprehensive recent analyses on data over much longer timescales (months to years; Maturana et al., 2020) indicate that critical slowing down may be evident prior to seizures at much longer timescales than the ones studied in the present paper. The authors at present do not cite or discuss these studies (refs. 1 & 3 above). I suggest that considerations about time window should be carefully discussed already in the introduction of the paper; conclusions made from the limited time window observations should be more cautious and take references (1) and (3) into account (see also next point for another concern that the authors do not discuss in their findings).

3. Lack of consideration of noise effects:

The authors do not consider the role of noise in the interpretation of their findings. Broadly speaking, critical slowing down can only be observed under moderate noise levels; if noise is too large, then it will cause a transition to the new state (crossing the bifurcation point) even if the bifurcation point is still relatively far away. The Maturana et al. 2020 manuscript includes a more detailed discussion of these matters as well as the more theoretical accounts on critical transitions provided in Kuehn 2011 (https://arxiv.org/abs/1101.2899). The authors may want to include a critical discussion of these noise aspects. For example, it may be conceivable that critical slowing down (i.e. the distance to criticality) is shaped on much longer timescales, such as days (Maturana et al., 2020) and the eventual seizure onset is then primarily driven by noise that pushes the system into the seizure state once it is sufficiently close to criticality.

4. A more balanced overview of the literature:

There a several statements in the manuscript that do not take into account other studies and the manuscript could benefit from being a bit more cautious in its conclusions by also giving a more broad, balanced account of the literature. Some statements may actually be a bit ill-fated or potentially even wrong in light of the literature that is not scanned appropriately and considered at present. Some examples (author statements set in italic):

Such inherent unpredictability is characteristic for critical systems, and may provide an explanation why reliable approaches for seizure prediction have not been established to date [33,34].

One may equally argue the opposite: the fact that seizure prediction is at all possible (Cook et al., 2013; and others) indicates that there are systematic changes in the neuronal signals that are being picked up by prediction algorithms (which could, in principle, be related to critical slowing down). I suggest to state this a bit more cautiously.

In the first scenario, one expects the seizure onset zone (SOZ) to generally operate closer to instability, and thus a clear difference between the SOZ and the same brain area in the conta-lateral, nonfocal hemisphere (nSOZ) (Fig. 1b).

This hypothesis, as stated, ignores a large body of research indicating epilepsy to be a network disease, where regions far from the SOZ may be more indicative of an upcoming seizure.

To quantify m, we made use of a novel, unbiased estimator that only requires knowing the number of spikes sampled from a small set of neurons to return a reliable estimate of the branching parameter m [36]. Importantly, the estimator is invariant under subsampling [37], thus it can infer the propagation of activity in a network even when recording only a small fraction of all neurons [12,38].

This estimator is essentially a fit parameter to the autocorrelation function of neuronal activity. The authors may consider citing other studies that have used the spiking autocorrelation function to infer distance to criticality (e.g. Bellay et al., 2015 Irregular spiking of pyramidal neurons organizes as scale-invariant neuronal avalanches in the awake state; Meisel et al., 2017 The interplay between long- and short-range temporal correlations shapes cortex dynamics across vigilance states).

For longer putative pre-seizure periods (30 minutes to 4 hours), signatures of 'critical slowing down', which are associated with transitions to supercriticality, have not been found either [32], which is consistent with our results.

Please consider references 1 and 3 above, which claim the opposite of what you write, and kindly balance your statement.

Thus for seizure prediction as early warning, which would be important for patients to seek a safe environment before a seizure starts, the framework of criticality has not proven useful yet.

Please consider reading Maturana et al., 2020, where the incorporation of critical slowing down into seizure forecasting algorithms has provided the best seizure forecasting algorithm to date. The above sentence is thus simply incorrect.

In line with this idea, the study that found signatures of critical slowing during seizure termination found these signatures only on the more coarse recording levels (EEG, ECoG, LFP), not in multi unit activity [30]. Therefore, the sparsely recorded single unit activity may not be the ideal type of signal to identify seizure onset dynamics.

As mentioned above, this important concern about the choice of methods should be emphasized at the beginning already as as it is essential to interpretation of all results.

In summary, we found no evidence for epilepsy to involve a transition towards supercritical dynamics

This statement, in its generality, may be misleading since the authors never present results from an analysis of ictal data. Ictal dynamics may or may not be supercritical, it is simply not being studied in the present manuscript.

5. A more comprehensive analysis of the autocorrelation estimator

The authors report no systematic change in the autocorrelation structure (assessed by their estimator) across cortical regions or across time. One may thus ask whether their estimator can confirm other findings reported in the literature in order to determine whether the indicator is robust/valid:

- For example: does the indicator show a change during seizures? It may be argued that seizure dynamics is very different from interictal dynamics, leading often to a complete or partial functional loss of the affected cortical region. Is the estimator able to capture such a change? Why did the authors not report on findings during seizures? This would be very interesting and seems like a missed opportunity. I understand that findings may vary widely and there may be no coherent picture emerging. Nevertheless, the manuscript might benefit strongly by including such an analysis, even if it means reporting non-coherent findings that vary across seizures and patients. Given the brevity of the study and the importance to assess what the estimator picks up during a seizure, I think an extension of the analysis to include ictal data would be crucial.

- Similarly, does the estimator show a change at the offset of the seizure, such as reported in Kramer et al., 2012?

- Does the estimator confirm a hierarchy of temporal scales, as reported e.g. in Murray et al., 2014 (also cited by the authors)? This work uses, very similarly, the autocorrelation function and has reported an increase in timescales with cortical hierarchy. The authors already provide subanalyses for different regions; could this be extended to compare regions with low and high levels of hierarchy?

I think these questions are important to better understand what the authors' indicator actually measures. If it measures the same values at all times and cannot confirm findings from other studies in the past, then one may question these earlier studies or, alternatively, be cautious about validity of the indicator used in the present study.

6. Exclusion criteria of MR estimation:

Could the authors please elaborate more on the impact of the the second exclusion criterion? As is well known, the autocorrelation function will decay geometrically at criticality. It can be argued that a power-law decay may look more like a linear decay than an exponential decay, particularly on short time lags. The linear fit may thus potentially be the better fit at criticality. Thus, requiring that the autocorrelation function must be better fit by an exponential than by a linear fit may impose a systematic bias towards subcriticality (where it decays exponentially)? Could the authors please elaborate?

7. The authors should please confirm and state explicitly that, when comparing different cortical regions in one patient, data was collected from the same time intervals, as it is known that the autocorrelation structure of neural signals is also strongly influenced by circadian rhythms and AED action (e.g. ref. 47).
