# Supplementary material for: Assessing criticality in pre-seizure single-neuron activity of human epileptic cortex
Source: PLoS Comput Biol. 2021 Mar 8;17(3):e1008773. doi: 10.1371/journal.pcbi.1008773 (PMC7971851; doi:10.1371/journal.pcbi.1008773)
Supplement: Attachment — Submitted filename: replies.pdf [file pcbi.1008773.s012.pdf]

Response to reviewer 1

Reviewer 1: *Hagemann et al. present in their paper an analysis of spatial and temporal changes in "criticality" of groups of single-neuron activity in the human brain, but claim that these changes are not related to the brain's proximity to epileptic activity (spatially or temporally). I have some comments which I hope will help the authors improve their paper.*
I have two main issues with this paper, which may simply be a matter of clarification, but definitely have implications for the interpretation of the paper by the wider scientific community.

1) *As far as I can see, you do not distinguish between spikes from excitatory and inhibitory neurons. Your Fig. 1a only makes sense if all your spikes are excitatory. If you really do no distinguish the two types of spikes in the data, then I do not think you can claim the interpretation on the branching parameter as you do. M=1 is not necessarily the critical point. Please either clarify and/or provide simulations of networks of excitatory and inhibitory neurons to show the interpretation of the branching parameter.*
This is indeed a valid point, we do not distinguish excitatory and inhibitory connections. Instead, we use the branching parameter m to describe the *effective* spreading of activity in the network. This becomes most clear when considering a small perturbation (spike) to the network. The branching parameter describes how many spikes are, on average, triggered by such an additional spike – incorporating effectively the underlying neuron types, network topology, and excitatory/inhibitory connections. If e.g. an inhibitory neuron is activated, which in turn inhibits an excitatory neuron which would fire otherwise, then this is incorporated into the effective m. Averaging over all these aspects (by not distinguishing excitatory and inhibitory connections), and assuming sufficiently small perturbations, $m = 1$ would be the critical point. To support this approximation, Wilting VP, Cerebr. Cortex 2019 showed that the branching process model does indeed reproduce a number of statistical properties of cortical networks in vivo, which clearly have excitatory and inhibitory contributions. We added this aspect to the figure caption and the discussion:
Caption: Both excitatory and inhibitory connections are summarized to an *effective* excitation.
Discussion: Our estimation of the distance to criticality relied on the branching process approximation. Mathematically, the branching process represents a generic model of how activity propagates in a network, but clearly, it is quite simplistic, and does not account for all the biological complexity in the brain. In particular, we do not distinguish excitatory and inhibitory connections. Instead, we use the branching parameter m to describe the *effective* spreading of activity in the network. Any alteration in m may thus reflect one or a combination of mechanisms, like altered synaptic strength, excitability, or excitation-inhibition ratio. Despite this simplification, a previous study has shown that the branching process model does indeed reproduce statistical properties of networks with inhibition, if one assumes that the excitatory and inhibitory contributions can be described by an *effective* excitation [12].

2) *I find your definition of SOZ very vague and misleading. As far as I understand it, you assign spikes as SOZ if they come from the hemisphere of the seizure focus (are these patients all MTLE patients?). This is a gross oversimplification. You could simply give it a different name to "SOZ" (which has a clear clinical definition in epilepsy), but there is still a fundamental interpretation problem: all your spikes could have come from a region that is unrelated to the seizure-generating part of the brain. The only gold standard for "epileptogenicity" is by confirming it through epilepsy surgery and showing that the patient is seizure free afterwards. The clinically-defined seizure onset zone is a working approximation to the gold standard. You need to be very clear in your paper about these concepts. Please rename your SOZ, nSOZ; provide additional analysis of spikes from the confirmed clinically-defined SOZ vs. other spikes. If surgery location and outcome is known, please provide additional analysis on spikes that come from surgically removed areas in patients that are seizure-free after surgery.*

Thank you for pointing this out. We agree that renaming the locations as "focal" and "contra-lateral" is more precise. Indeed, all patients were MTLE patients, and we used their electrographic focus. As we record from areas within MTL only, the SOZ is in our case very similar (large overlap) to the brain areas we record from. 10 out of the 20 patients had an ideal surgery outcome (Engel scale 1a). We now added the surgery outcomes of all patients to table S1 in the Supplementary. As suggested, we now also added the main analyses using only data from these 10 confirmed patients (see Figs. 1, 2):
So far, we have used the *electrographic* focus for our analyses, i.e. the brain area in which seizures emerged, determined by clinicians prior to surgery. However, surgery outcomes of the patients differ (see Table S1). We therefore ran the main analyses again, including only patients that were seizure-free after the surgery (Engel scale 1a, 10 out of 20 patients). Consistent with our previous findings, we found no significant change before seizure onset in these patients (Fig. S8). Furthermore, we found no significant differences between focal and contra-lateral hemispheres neither in the full MTL, nor in the individual subregions (Fig. S7). In particular, the previously observed increased distance to criticality in focal hippocampus was not present in this reduced dataset. Note, however, that the number of recordings was smaller compared to the full dataset, such that the required effect sizes for significant results were correspondingly larger.

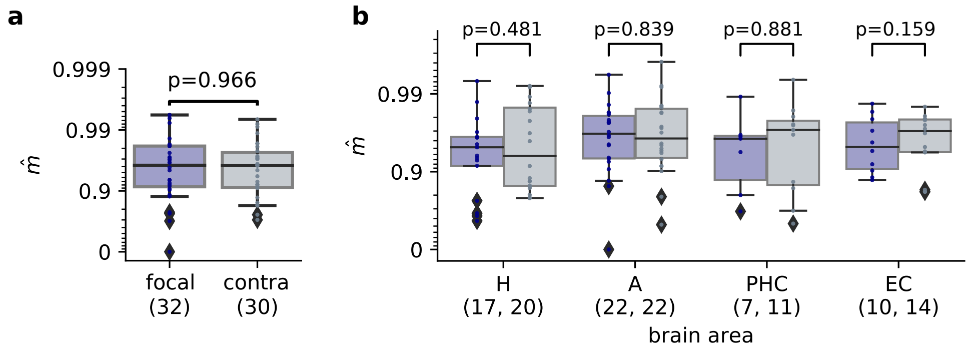

Fig 1. This figure corresponds to Fig. S7 in the supplementary of the paper. Comparison of focal versus contra-lateral hemisphere using only recordings from the 10 patients with 1a outcome after surgery. **a** The branching parameter $\hat{m}$ across recordings and patients shows no significant difference between focal and contra-lateral hemisphere. **b** Comparison of the branching parameter $\hat{m}$ in focal and contra-lateral hemisphere for different subregions of the MTL, (hippocampus (H), amygdala (A), parahippocampal cortex (PHC) and entorhinal cortex (EC)). None of the regions shows a significant difference between focal and contra-lateral hemisphere ($p < 0.05/4$ required after Bonferroni correction).

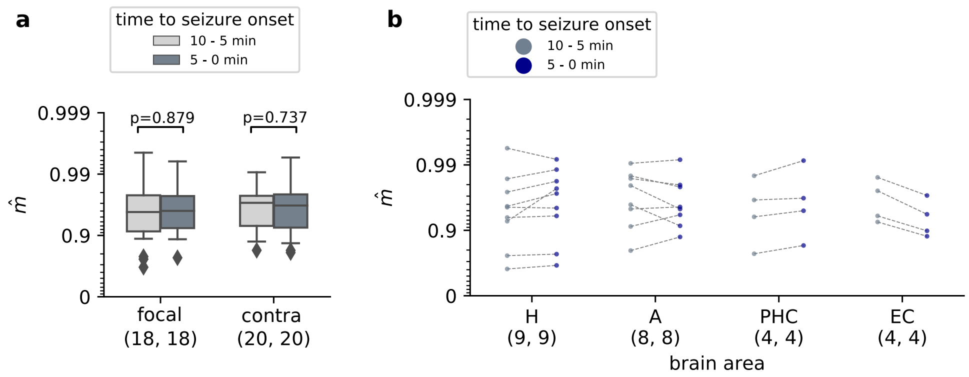

Fig 2. This figure corresponds to Fig. S8 in the supplementary of the paper. Analysis of pre-seizure changes using only recordings from the 10 patients with 1a outcome after surgery. **a** No significant difference in the distance to criticality between the last 5 min prior to seizure onset and the previous 5 min – neither in the MTL containing the epileptic focus, nor in the contra-lateral one (p-values of Wilcoxon signed rank test). **b** Separate analysis of the individual subregions of MTL in the hemisphere containing the epileptic focus. Due to the small number of rescordings, we report the individual estimates without performing statistical tests.

Furthermore, I have some issues with the technical and statistical aspects of this work. In no particular order:

3) *The spike sorting is not sufficiently described, what types of electrodes were used, I presume micro-wires? How was it determined where the microwire is located exactly for the SOZ analysis and also the brain area analysis? How does the number of microwires used for each brain area (I presume some contacts needed to be removed due to noise) affect the m estimations?*

To clarify the data acquisition and spike sorting approaches, we expanded the methods section in the manuscript: For pre-surgical evaluation, patients were implanted with depth electrodes in different regions of the medial temporal lobe, including hippocampus (H), amygdala (A), parahippocampal cortex (PHC) and entorhinal cortex (EC). The location of microwires was verified using post-implantational CT scans co-registered to pre-implantational MRI scans. All wire bundles were confirmed to be located in the designated target regions in each patient. Each electrode contained 8 microwires, with which single-unit recordings could be preformed. [...]
Spike sorting was performed using the Combinato package [64], using the standard parameters proposed by the authors. Sorted units were classified manually as single units, multi-units, or artifacts, using the Combinato GUI [64]. The main classification criterion for putative units was the signal's shape, but also the amplitude and distribution of inter-spike-intervals, which allows excluding artifacts due to e.g. supply voltage. For further analysis, we only used spikes of identified single units and excluded artifacts and multi-units. By focusing our analysis on single-units, and excluding putative multi-units, we minimized the number of artifacts, which can potentially impact subsequent analyses.

Regarding the question of how the number of microwires (or single units) used for each brain area affects the m estimation: Provided that we have sufficient putative single neurons such that the mean field approximation applies, the number of microwires (and thereby the number of recorded neurons and spikes) will only affect the variance in estimates. The lower the spike count, the higher the variance (see Suppl. Fig. S4). In that figure, we reduced the recording length, but equivalent results are expected when reducing the number of neurons instead, as shown in Wiling & Priesemann, 2018.

4) *You claim m is "unbiased by undersampling": do you expect m to represent a local neural population? A brain area? A whole hemisphere? This will presumably depend on how much tissue the microwires are sampling?*
This is an interesting conceptual questions. In principle, the branching parameter m can characterize a network of any size, in which neurons are recurrently connected and that is sufficiently homogeneous. In practice, however, it is indeed a very reasonable question, which parts of the brain can be considered sufficiently homogeneous; this may even depend on cognitive state and task. A local population can certainly be characterized by a branching parameter m. However, these local branching parameters can differ depending on the brain area: studies on intrinsic timescales, which are directly related to m, have shown that different brain areas can have different intrinsic timescales (e.g. Murray et al. 2014). Thus, when reporting the branching parameter of a full hemisphere, this must be interpreted as an average of the branching parameters of the subregions in which neurons were recorded.
As we only analyze data from the MTL and not the entire hemisphere, potential differences between brain areas do not affect our results too much, which is supported by the fact that we do not find systematic differences when leaving out individual subregions of MTL (see sensitivity analysis Fig. 3 in the replies). Furthermore, even when separately analyzing the subregions of MTL, we find no significant change when approaching seizure onset (Fig. 2c in main text). We therefore also report m over all recorded neurons in MTL, as it characterizes the dynamics across MTL and enables a more highly resolved temporal analysis.

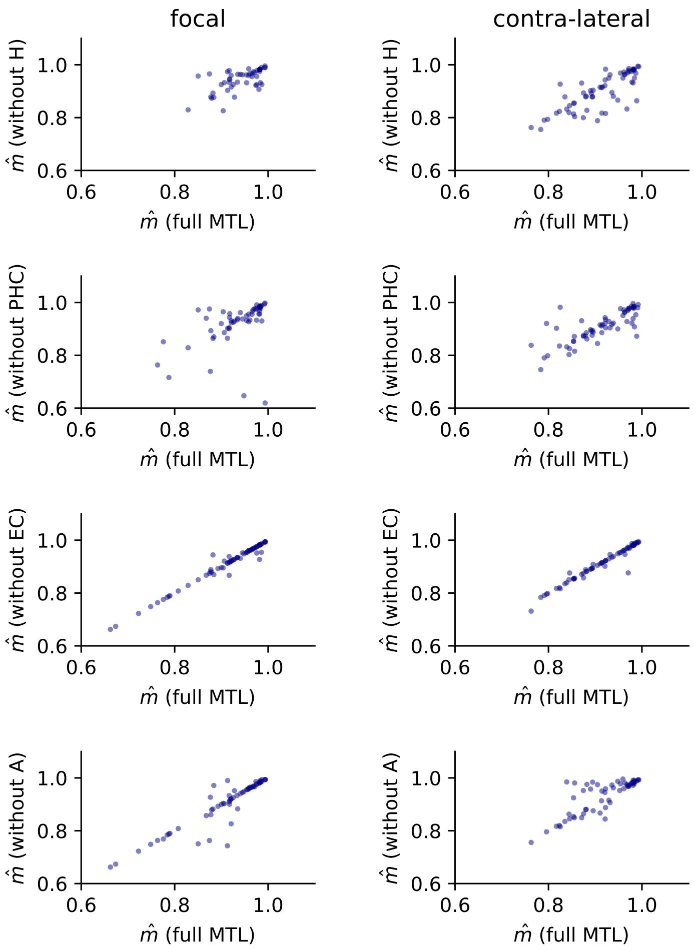

Fig 3. Estimated branching parameters of the MTL when removing one of the subregions, shown both for the focal and the contra-lateral hemisphere. Note that recordings, in which no estimation is possible after removal, are not shown. This can occur if the remaining number of spikes after removal is too low for reliable estimation.

5) *How much do spike sorting parameters affect m?*

Spike sorting parameters will influence how restrictive the selection of potential spikes is performed. Restrictive spike sorting will result in a lower number of accepted spikes, while less restrictive spike sorting will result in a higher number spikes, but also a higher number of artifacts. We used the Combinato package and with standard parameters [64]. We decided to perform spike sorting restrictively, by using only identified *single units*. Thereby, we minimize the number of artifacts, which could impair branching parameter estimation (especially temporally correlated artifacts would affect m). As the MR estimator is invariant under subsampling, a lower total number of accepted spikes without artifacts is preferable over a large number of spikes with artifacts.

6) *In the temporal analysis, is it really fair to combine all A_t to represent one hemisphere? Given that some brain regions were missing in some patients I'd like to see a sensitivity analysis of m estimates for a hemisphere with and without e.g. one (two) brain region(s).*

Thank you for this input. It also touches the question of which fraction of the brain the branching parameter m can characterize (question (4)). Fig. 3 shows a sensitivity analysis, i.e. estimates of the branching parameter using the full MTL vs. estimates without one region. It shows that that m does indeed fluctuate when leaving out a brain region – however, these fluctuations are not systematic, but go into both directions for all regions.
Furthermore, the temporal analysis mainly aims at detecting changes before seizure onset. Even though some regions are missing in some patients, these regions will be missing across the full 10 min recording, thereby not affecting the detection of changes. It is conceivable that only a subregion changes before seizure onset, while other regions overshadow or even compensate this effect. The more coarse temporal analysis, that we performed separately for all regions, however, does not indicate this is the case (Fig. 2c in main text).

7) I would be interested in seeing equivalent results for Fig 1d&f & Fig 2b,c but using a hierarchical model/analysis where subject is modelled as a random effect. Systematic differences between subjects is possible, especially given that m=1 may not represent a universal criticality threshold depending on sampling.
Thank you for this input. We agree that modeling patientID as a random effect, not a fixed effect is reasonable, as those patients are themselves a sample of the whole population of MTLE patients. Instead of the ANOVA for Fig. 1 in which patientID was a fixed effect, we now computed the ANOVA with patientID as a random effect, using the lmer function by R (more precisely, using its python interface with pymer4). Furthermore, we performed the respective pairwise comparisons taking into account patientID as a random effect. We now find a just significant difference between focal and contra-lateral hemisphere in hippocampus. Contrary to our hypothesis, the focal hippocampus seems to operate further away from criticality. The effect is at the margin of significance, but we added a short discussion paragraph on this finding.
For Fig. 2b&c, where we compare the first with the second halfs of pre-seizure recordings, we always have *paired* data. The statistical tests should therefore not be affected by potential differences in the absolute values of m between patients (the test only measures the pairwise differences between first and second halves). We therefore consider the non-parametric paired Wilcoxon test the best choice for this analysis. As an additional sanity check, we nevertheless performed the hierarchical analysis for this data as well, and the results were consistent.
The new analysis has lead to multiple changes in the manuscript, here the mayor ones:

New methods concerning Fig. 1: For each recording, the spikes from the hemisphere containing the epileptic focus were combined and binned to a single activity time series A_t^{focal}. Equally, all recorded spikes from the contra-lateral hemisphere were binned to A_t^{contra}. Estimated branching parameters $\hat{m}$ of focal and contra-lateral hemisphere were compared by performing a mixed effects ANOVA, taking into account the patientID as a random effect. As the distribution of $\hat{m}$ was not consistent with a normal distribution, we performed the mixed effects ANOVA on the logarithmic distance to criticality $\hat{\epsilon} = \log(1 - \hat{m})$. Specifically, we used the python interface *pymer4* [66] to the R-function *lmer* [67], with model specification $\log(1 - \hat{m}) \sim \text{focus} + (1|\text{patientID})$. As differences in between patients are possible, we additionally compared focal and contra-lateral hemisphere separately within each of the patients.
The same approach was applied individually to each subregion of the MTL. In this case, spikes were binned separately for each subregion in each hemisphere. To disentangle potential effects of patient-ID, subregion and location of the epileptic focus, we conducted a mixed effects ANOVA, where patient-ID was modeled as a random effect. As above, we used the python interface *pymer4* [66] to the R-function *lmer* [67], with model specification $\log(1 - \hat{m}) \sim \text{focus} \cdot \text{subregion} + (1|\text{patientID})$. In addition, we performed post-hoc pairwise comparisons of $\hat{m}$ of focal versus contra-lateral hemisphere in all subregions using the model specification $\log(1 - \hat{m}) \sim \text{focus} + (1|\text{patientID})$.

New results concerning Fig. 1: We tested the hypothesis that the focal hemisphere generally operates closer to the critical point, which would make it prone to tipping over to unstable dynamics (Fig. 1b). However, we found no significant difference between focal and contra-lateral hemisphere for $\hat{m}$ across recordings and patients (mixed effect ANOVA with patientID as random effect, $p \approx 0.84$). *Within* some of the patients, however, there were consistent differences in the distance to criticality between hemispheres (Figs. 1e, S2). Out of the 8 patients for whom there were sufficient recordings for a significance test, we found $\hat{m}_{focal} < \hat{m}_{contra}$ in 2 patients, $\hat{m}_{focal} > \hat{m}_{contra}$ in 2 patients and no significant difference in the remaining 4 patients. Our result thus suggests that there can be consistent differences in distance to criticality between hemispheres but that the location of the epileptic focus does not predict that difference.

To test whether the distance to criticality in the focal hemisphere is only altered in specific subregions of the MTL, we analyzed the recorded activity A_t separately in each of the subregions, including amygdala, entorhinal cortex, parahippocampal cortex and hippocampus. Consistent with the above results, we found that single unit activity in all subregions reflects a subcritical regime (Fig. 1f). To test for differences between focal and contra-lateral hemisphere, we jointly took into account the effects of brain area, location of the epileptic focus and patient-ID. We found a significant interaction effect of brain area and focus (mixed effect ANOVA with patientID as random effect, $p \approx 0.021$). The post-hoc comparisons of the distance to criticality within each subregion revealed a significant difference between focal and contra-lateral hemisphere in hippocampus (mixed effect ANOVA with patientID as random effect, $p \approx 0.012$). However, in contrast to our expectation, the results indicate that the hippocampus of the focal hemisphere operated further away from criticality than the contra-lateral hippocampus. In all other subregions, we found no significant differences between focal and contra-lateral hemisphere (Fig. 1f).

New discussion paragraph concerning Fig. 1: Interestingly, we found that the branching parameter in the hippocampus of the focal hemisphere was smaller than in the contra-lateral hippocampus. This contrasts our initial hypothesis of a reduced distance to criticality in the focal hemisphere. A potential explanation for the increased distance to criticality could be the destruction of neuronal tissue (e.g. mesial temporal sclerosis), which is frequently observed in MTL epilepsy [52]. Neuronal cell loss can result in a more segregated network, reducing the effective coupling between neurons and therefore the branching parameter. Another potential reason are compensatory mechanisms that reduce the coupling strength between neurons because of repeated excessive activity in that area (e.g. via homeostatic plasticity).

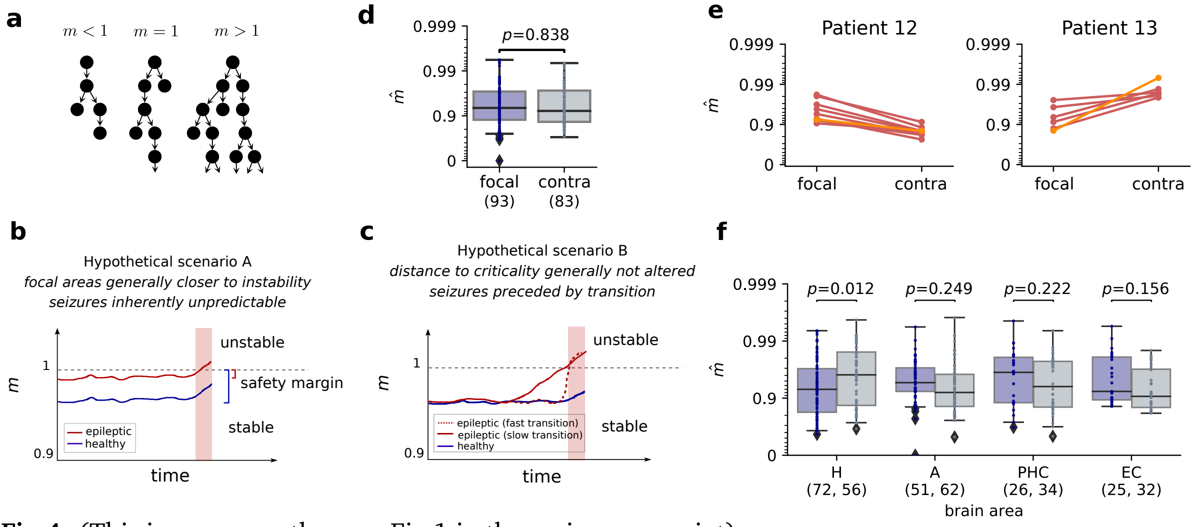

Fig 4. (This is a copy on the new Fig.1 in the main manuscript)
Comparison of the distance to criticality between the hemisphere containing the epileptic focus and the contra-lateral hemisphere. a Branching process approximation of activity propagation in the brain. Both excitatory and inhibitory connections are summarized to an *effective* excitation. Depending on the branching parameter m, dynamics are stable ($m < 1$), unstable ($m > 1$) or critical ($m = 1$). **b, c** Hypothetical scenarios of seizure generation. The epileptic focus might, in general, operate closer to criticality and consequently enter the supercritical regime in an unpredictable manner because of small fluctuations (scenario A). Alternatively, dynamics might systematically drift towards instability before seizure onset. If that drift is sufficiently slow, seizures might be predictable (scenario B). **d** The branching parameter $\hat{m}$ across recordings and patients shows no significant difference between focal and contra-lateral hemisphere (mixed effect ANOVA with patientID as random effect). **e** Estimated $\hat{m}$ in focal and contra-lateral hemisphere for multiple recordings of two exemplary patients. While several patients showed a consistent difference between hemispheres across recordings, this difference is not predicted by the location of the epileptic focus (orange: reference recording, red: pre-seizure recording, see Fig. S2 for all patients). **f** Comparison of the branching parameter $\hat{m}$ in focal and contra-lateral hemisphere for different subregions of the MTL, (hippocampus (H), amygdala (A), parahippocampal cortex (PHC) and entorhinal cortex (EC)). The hippocampus shows a just significant difference between focal and contra-lateral hemisphere, with a smaller m in the focal hemisphere. The other subregions show no significant difference ($p < 0.05/4$ required after Bonferroni correction). Box plots show median and quartiles, while whiskers extend to the rest of the distribution, except for outliers (points beyond 1.5 x interquartile range).

7) What type of seizures were included in this analysis – subclinical & clinical seizures?

Only clinical seizures were included in the analysis. We added this information to the methods section.

Response to reviewer 2

Reviewer 2: *In the submitted manuscript No evidence that epilepsy impacts criticality in pre-seizure single-neuron activity of human cortex.", Hagemann et al. analyzed single-unit recordings obtained in patients with epilepsy to test whether 1) epileptogenic tissue operates in a highly unstable regime close to the critical point of bifurcation, and 2) seizures are preceded by progressive changes in the bain stability. To estimate the stability/instability, they used an estimator that they developed and successfully applied to various datasets, including spike train data. The method estimates the branching parameter that reflects the spreading of neuronal activity.*
Firstly, they determined the stability inside the SOZ zone and outside the SOZ. They did not find any differences between these regions. Current and previous findings suggest that the human brain operates physiologically close to the critical point and that epileptogenic tissue does not display higher instability (decreased stability) than the tissue outside SOZ. Secondly, the authors did not reveal any consistent changes in cellular activity that would be indicative of increasing instability with approaching seizures. The article is focused, and the results are worth publishing. It brings an important piece of information about the cellular dynamics involved in seizure genesis and the dynamical properties of epileptogenic tissue. The discussion is written quite well. The authors critically discuss the weaknesses of a single cell approach, and they critically evaluate obtained results with the existing literature. The manuscript is interesting and evokes many questions. Some of them should be addressed in the revised version of the article.

1) *The authors should justify in greater detail the reasons for the selection of the branching estimator to determine the stability/instability. The methods estimates activity spread. Did the authors assume that the epileptogenic tissue would be characterized by a higher probability of cell recruitment or stronger connectivity between neurons? Does the hypothesis suggest that with approaching seizure, cellular recruitment will increase? Some studies suggest that spatial expansion marks the system's decreasing resilience and distance from the bifurcation (Scheffer at al, Nature, 2009).*
Thank you for pointing this out. The rationale behind using the branching parameter to characterize stability is that uncontrolled, excessive neuronal activity can arise if neurons are abnormally excitable (altered excitation-inhibition ratio), or if circuits of neurons are abnormally connected [40]. Both would result in more correlated neuronal activity and can therefore be captured by the branching parameter m. Furthermore, independent of the branching process model, the branching parameter is an indicator of *critical slowing*, which is expected when a system approaches a critical transition [25].
We now added these aspects to the introduction:
[...] By characterizing activity spread, the branching parameter incorporates multiple network properties, whose alterations may be associated with epilepsy, including the excitability of neurons, the excitation-inhibition ratio, and the connectivity [40, 41]. More generally, an increasing branching parameter indicates more temporally correlated neural activity [12] and is therefore a signature of *critical slowing*, which is expected when a system approaches a critical transition [25].

2) The results of current and previous studies claim that a healthy human brain operates in a highly unstable regime. Such a generalization would suggest that even a healthy brain is prone to seizure genesis?
In our view, working point of healthy cortex is not in a highly unstable regime, but within the stable regime, though close to instability. A lack of fine-tuning could then lead a transition to instability. If you refer with the term "highly unstable" to the hypothesis might operate close to the critical transition with only a fairly small safety-margin, then sure, we agree.
As epilepsy patients experience seizures regularly, and often without specific trigger, whereas healthy humans only very rarely, in extraordinary situations (the healthy brain is less prone to seizures), a qualitative difference between healthy and epileptic cortex could be expected. Our working hypothesis, when starting the study, was that this difference lies in the distance to criticality: while healthy brains maintain

a safety margin to instability, this safety margin might be generally reduced, or diminish systematically before seizure onset, in epilepsy patients. However, our findings show no evidence that the epileptic brain operates closer to criticality or drifts to criticality before seizure onset. There are different possible explanations for this negative finding (see discussion). However, we would not conclude that cortex is generally operating in a highly unstable regime.

3) How is the level of estimated stability influenced by circadian rhythm, sleep, sleep stages, or levels of vigilance?

The influence of circadian rhythm and sleep stages on the distance to criticality is indeed an interesting question. Studies on human epilepsy patients suggest that indicators of critical slowing can change with circadian rhythm (Wilkat Lehnertz, 2019) and sleep stage (Priesemann et al., 2013). Likewise, the very recent study by Maturana et al.,2020 indicated such changes. Furthermore, the branching parameter m is based on the autocorrelation structure of the neuronal activity, which was shown to change with vigilance state (Meisel et al., 2017).

We assume that the influences of circadian rhythm and sleep stages affect both hemispheres equally, and therefore will not affect our results significantly. To exclude that recordings of one hemisphere, of which we had no corresponding recording in the other hemisphere, bias the comparison, we now also added the results of Figure 1, but using only *paired* recordings in same time intervals (Fig. 7). We added the following text:

The autocorrelation structure of neuronal activity, and therefore the branching parameter m, can change depending on vigilance state and circadian rhythm [45, 35, 37, 46]. Furthermore, anti-epileptic drugs modulate cortical excitability and were shown to affect the branching parameter [47, 48]. To make sure that temporal differences do not overshadow differences between the focal and the contra-lateral hemisphere, or wrongfully give rise to apparent differences, we performed the same analyses using only *paired* recordings (recordings obtained from the same patients in the same time intervals). Consistent with the results shown in Fig. 1d,f, we found a significant difference between the focal and the contra-lateral hemisphere in hippocampus, and no significant differences in the remaining subregions and the full MTL (Fig. S3).

4) The authors analyzed data from patients who underwent presurgical evaluation. Often, the antiepileptic medication is tapered at the beginning of the intracranial recording. At its end, the medication is introduced again. Is it plausible that drug withdrawal increases instability (Zijlmans et al., Neurology, 2009)? In such a scenario, the pre-ictal changes may be absent because the brain dynamics is very close to the bifurcation. The distance to the bifurcation would be minimal, and the transition to seizure very fast (basically unobservable).

Indeed, studies suggest that drug withdrawal may increase the branching parameter (e.g. Meisel 2020). This would, in fact, be a potential reason of why one cannot detect pre-ictal changes. However, the branching parameter that we measured was, on average, not significantly higher than the branching parameter in other healthy mammalians (in fact rather smaller) (see Wilting et al. 2018 for other mammalians). Thus, unless the human MTL generally operates at a lower branching parameter than other mammalians, we find no indication that our patients were, in general, already unusually close to instability.

Nevertheless, a fast transition, that is potentially only driven by noise, is a potential alternative route to seizures and we now added it into the discussion:

Or the transition could occur on a much slower time scale, such that changes during the last 10 minutes are too small to be detectable. Once the system is sufficiently close to criticality, it could spontaneously be driven into a seizure by noise. This hypothesis is supported by a recent study that found signatures of critical slowing on a timescale of hours to days before seizure onset in human iEEG [35].

Furthermore, we added the aspect of antiepileptic drugs:

Furthermore, anti-epileptic drugs modulate cortical excitability and were shown to affect the branching

parameter [47, 48].

5) The authors did not observe a drift in instability ahead of seizures. Apart from analyzing a trend, the authors could explore the fluctuations in the branching parameter in advance of seizures
We agree that fluctuations in the branching parameter could, in principle, also be an indicator for an impending seizure. In fact, it could be argued that strong fluctuations in the branching parameter can lead to a spontaneous tipping over to the unstable regime. We now added an analysis of the variability of $\hat{m}$, but did not find evidence for systematic differences (Fig. 2d in the main manuscript, copied also below).

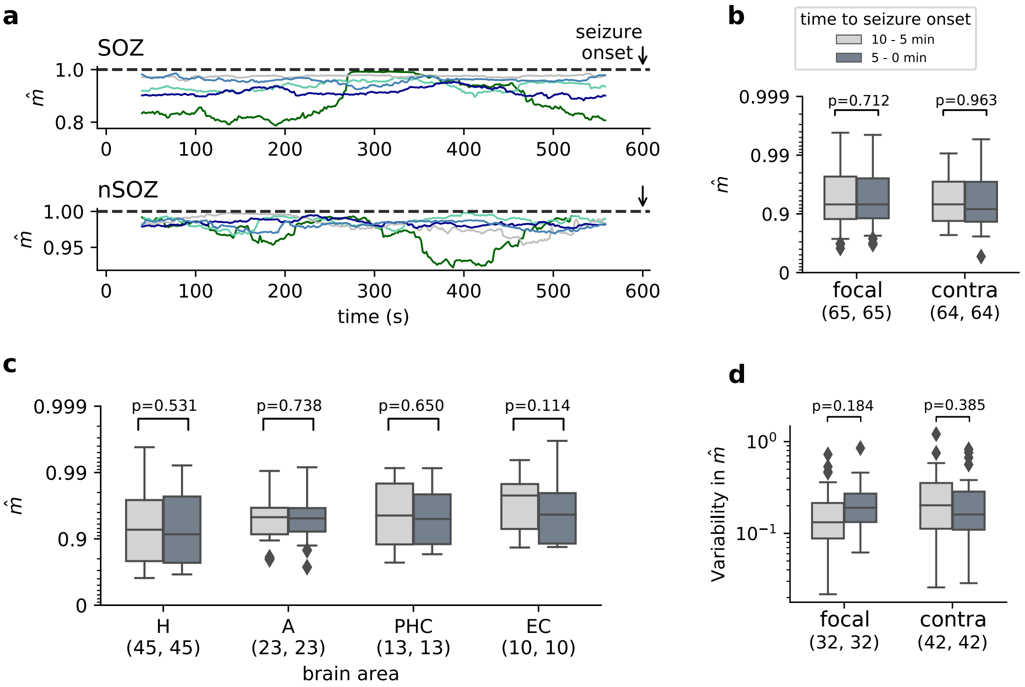

Fig 5. (Copy of Fig. 2 of the main manuscript)
No consistent change in the distance to criticality before seizure onset. a Time-resolved estimation of $\hat{m}$ for the full MTL within the last 10 min prior to seizure onset showed variability, but no consistent behavior during the last minutes before seizure onset. Plots show example traces of multiple seizures from one patient. Each trace corresponds to one pre-seizure period, shown both for the focal and the non-focal hemisphere. Traces of all patients and seizures shown in Fig. S6. **b** No significant difference in the distance to criticality between the last 5 min prior to seizure onset and the previous 5 min – neither in the MTL containing the epileptic focus, nor in the contra-lateral one (p-values of Wilcoxon signed rank test). **c** Separate analysis of the individual subregions of MTL. Within none of the subregions there were significant differences between the last 5 min before seizure onset and the previous 5 min. Box plots in **c** show results from the hemisphere containing the epileptic focus. **d** Variability of the time-resolved branching parameter $\hat{m}(t)$ in the last 5 min prior to seizure onset and the previous 5 min. Variability is quantified using the median absolute deviation from the median logarithmic distance to criticality (p-values of Wilcoxon signed rank test).

6) In this or future studies, the authors could use antiepileptic drug withdrawal to evaluate the estimator. Or vice versa, they could assess the effect of antiepileptic drugs on brain stability.
This is indeed an interesting idea. In fact, there is a relatively recent study that specifically looked at the

effect of anti-epileptic drugs on the branching parameter [47]. They show that antiepileptic drugs lead to more short-ranged brain dynamics in terms of spatiotemporal activity cascades and temporal correlations – which would be consistent with more subcritical dynamics. The study was based on epileptic discharges in ECoG data, and would indeed be interesting to verify their findings in single-unit data in future. We added the reference to our manuscript:
[...] Furthermore, anti-epileptic drugs modulate cortical excitability and were shown to affect the branching parameter [47, 48].

7) Seizure onset, progression, and termination can follow a different dynamical pathway between patients (Jirsa et al., Brain, 2014; Saggio et al., J. Math. Neurosci., 2017). Even in individual patients, multiple seizure types can exist, each with a distinct seizure onset and dynamical profile. The authors should discuss their results from this perspective.
Thank you for pointing out this aspect. It is indeed important for our discussion, as it provides one explanation for the variability of results across patients. We added this aspect to the discussion:
In addition to the variety of proposed seizure generating mechanisms, there is strong evidence that seizure onset, progression, and termination can follow different dynamical pathways [63, 33, 56]. Such differences can exist not only between patients, but also within individual patients. This renders finding a common mechanism or warning signal inherently difficult and may provide a further explanation of why we find no consistent effect within and across patients.

8) Previous studies have already proposed and demonstrated various pathways to seizure. The seminal work of Lopes da Silva should be cited (Lopes da Silva et al., Epilepsia (Suppl 12), 2003). Lopes da Silva and his colleagues proposed three possible trajectories to seizure onset from 1) stable state, 2) unstable state, and 3) via a slow drift towards the bifurcation. The work demonstrating high instability in photosensitive epilepsy should also be discussed and cited.
Thank you for pointing out the work of Lopes da Silva et al. on the different routes to seizures. We now added it to our introduction.

9) The pre-ictal changes in the stability and resilience were shown by Chang et al., Nat Neurosci, 2018. The results on the cellular dynamics should be compared with the observations in this study.
Thank you for pointing out the study of Chang et al., we now added it to our discussion of the pre-seizure analysis, see next point (10).

10) Recent studies demonstrate the existence of long-term fluctuations in seizure probability. Multiple rhythms can co-exist (circadian, multidien, multimonth) in each patient (Baud et al., Nat Commun, 2018; Karoly et al. Lancet Neurol. 2018). It is plausible that the process of the transition to seizure can vary between the periods of low and high seizure probability. These studies also suggest the combining the information from the multiple rhythms is highly informative about the seizure occurrence or that the slow changes in resilience can be observable at a long time scale (Chang et al., Nat Neurosci, 2018). The process of the transition to seizure occurring at the scale of minutes or hours can bring only partial information or no information at all.
Thank you for this comprehensive input. We agree that our analysis of changes prior to seizure onset is limited in terms of the considered time window. Especially for seizure prediction, it would be highly interesting to include longer time periods of days into the analysis. Such long-term spike recordings are, unfortunately, difficult to obtain. The rationale behind analyzing the 10-minute period is that, even though pre-ictal changes might occur on a slower timescale, they could be detectable in a 10-minute window (with an accordingly smaller effect size). Furthermore, we specifically analyze the *last* 10 minutes prior to seizure onset. Even if circadian, multidien and multimonth rhythms contribute, we would

expect some change in neuronal activity that can explain why the seizure occured at that specific moment, and not 10 minutes earlier (unless it is spontaneously driven by noise).
In future studies, our findings on the 10 minute time window can certainly be complemented by longer or extremely short periods. Nevertheless, our results provide important information on the currently accessible data and thereby provide a basis for future studies.
We extended the discussion of the considered time window, and added the aspect of different rhythms:
We found no evidence for systematic transitions from the sub- to the supercritical regime before seizure onset. As all our recordings have been obtained during seizure-free intervals (reference or pre-seizure), this finding does not contradict the experimental evidence that epileptiform activity and seizures proper might reflect a transition to the supercritical regime [27, 26]. In the literature, the reported time window of putative pre-seizure changes varies considerably from seconds to minutes and hours [53, 49, 54, 35, 30]. Even on longer time scales, recent studies suggest fluctuations in seizure susceptibility, including circadian, multidien and multimonth rhythms [55, 56]. Thus potentially, the transition to supercriticality happens on a time scale that is not detectable in the analyzed 10-minute interval. The transition could either be so rapid that our time-resolved analysis cannot capture it (faster than the 80 seconds analysis window). Or the transition could occur on a much slower time scale, such that changes during the last 10 minutes are too small to be detectable. Once the system is sufficiently close to criticality, it could spontaneously be driven into a seizure by noise. This hypothesis is supported by a recent study that found signatures of critical slowing on a timescale of hours to days before seizure onset in human iEEG [35]. A former study investigating putative pre-seizure periods of 30 minutes to 4 hours, on the other hand, did not find such signatures in human iEEG [37]. A recent study on ECoG data of human patients with focal epilepsy found clear systematic trends in signatures of critical slowing during the last 30 min before seizure onset, however these trends were not consistent across patients (increase in the signal's autocorrelation coefficient (ACC) in 4/12 patients, decrease in 4/12 and no significant change in the ACC in 4/12 patients) [30]. Thus it remains to be investigated precisely, under which conditions (e.g. types of epilepsy, types of recordings, considered time window) the framework of criticality can serve to predict an impending epileptic seizure.

11) The title should be modified because epilepsy does not play a causal role in the process.
We replaced the title to a version that does not imply causalities:
Assessing criticality in pre-seizure single-neuron activity of human epileptic cortex

Response to reviewer 3

The authors present a compact study which assesses the spike autocorrelation function during preictal and interictal periods and in different cortical regions. The study is motivated by the autocorrelation function's role in the context of critical transitions; the increasing timescale captured by the slower autocorrelation function decay when a critical transitions is approached is typically referred to as critical slowing down and may be used to infer the distance to criticality. Many epilepsy models assume a critical transition (or bifurcation) at seizure onset (see for example https://www.biorxiv.org/content/10.1101/2020.02.08.940072v1.full; also Jirsa et al., Brain, 2014) and the presence of critical slowing down prior to human seizures is currently a topic of debate and research. The present study touches on some of these timely questions by studying critical slowing down at the individual neuron dynamics near seizure onset. While the questions posed are interesting, the study unfortunately raises some methodological concerns and misses discussion of alternative explanations and key publications to put findings and their interpretation into a larger, more balanced context. The study may also benefit from a more comprehensive assessment that also includes ictal data and seizure offset. Nevertheless, the study is interesting and the use of individual neuron spiking and critical slowing down in epilepsy is novel. The authors should just extend their analyses to ictal data, be more cautious in their interpretations and provide a more balanced overview over the field (see comments below).
We thank you for this clear assessment. As a brief remark, we would be quite interested to extend our analyses to ictal data and seizure offset, however, these data are at the moment not available. However, we are happy to extend the analyses, and the discussion and interpretation. You find a point-by-point reply below.

Main concerns:
1. Choice of methods:
The authors study the autocorrelation structure of single neuron spiking activity to probe proximity to a critical transition. As highlighted by Kramer et al., 2012 (also cited by the authors) a critical transition may be present even though single neurons do not capture critical slowing down. As further shown in Kramer et al., dynamical signatures such as critical slowing down may be better accessible in data like LFP, EEG. In the present paper the authors conclude that critical slowing down is not present solely based on the study of single neuron dynamics, which consequently poses a dilemma whether the conclusions by the authors (that there is no critical slowing down prior to seizure onset) are justified. In fact, several papers, which are currently not cited by the authors, seem to suggest otherwise, i.e. that LFP and EEG do indicate some changes potentially in line with critical slowing down:
(1) Chang, W.C., Kudlacek, J., Hlinka, J., Chvojka, J., Hadrava, M., Kumpost, V., Powell, A.D., Janca, R., Maturana, M.I., Karoly, P.J., Freestone, D.R., Cook, M.J., Palus, M., Otahal, J., Jefferys, J.G.R., Jiruska, P., 2018. Loss of neuronal network resilience precedes seizures and determines the ictogenic nature of interictal synaptic perturbations. Nat. Neurosci. 21 (12), 1742–1752 12.
(2) Meisel, C., Kuehn, C., 2012. Scaling effects and spatio-temporal multilevel dynamics in epilep- tic seizures. PLoS One 7, e30371.
(3) Maturana M. et al. Critical slowing down as a biomarker for seizure susceptibility.
https://www.biorxiv.org/content/10.1101/689893v1 (paper is currently in print at Nat Comm 2020; DOI: 10.1038/s41467-020-15908-3).
I would suggest that, at the least, this dilemma, that single neuron activity may not be informative, as indicated by prior studies (e.g. Kramer et al., 2012), should be communicated transparently already in the introduction. Also, the authors should definitely provide a more balanced account on the literature citing also studies which may be more in line with indication of critical slowing down to balance their reporting. Currently, throughout the paper the authors predominantly only cite studies that did not find indication for critical slowing down at seizure onset, as for example: ...recent studies on seizure initiation did not find consistent warning signals of a critical transition prior to seizures [31,32]
Thank you for the comprehensive feedback. We agree that one potential limitation is that single neuron activity may not be informative about critical slowing (Kramer et al., 2012). We therefore wrote a

detailed paragraph in the discussion to communicate this issue. Note, however, that despite the study by Kramer et al., it is not clear that single unit recordings are not suited for inferring the distance to criticality. In fact, in a parallel study we find indications that spike recordings can be better than coarse recordings, such as LFP or EEG, when inferring the distance to criticality (Pinheiro et al. arxiv, 2020). That said, we totally agree that it is an essential point for the discussion, and therefore expanded on it. We now highlight this aspect again in the conclusion and added the references you pointed out to the introduction and discussion:

The conclusion highlighting the single-unit aspect again now reads as follows: In summary, we found no evidence for a transition towards supercritical dynamics in pre-seizure single neuron activity of human cortex. This finding is in line with previous studies finding no warning signals of a critical transition prior to epileptic seizures [37, 36], but stands in contrast with a recent study demonstrating the effectiveness of signatures of critical slowing for seizure prediction [35]. It remains to be investigated precisely whether single-unit recordings, despite providing the most direct information on neuronal activity, cannot resolve pre-seizure changes in criticality.[...]

The introduction paragraph with the additional studies now reads as follows: Epileptic seizures have been hypothesized to reflect a transition to supercritical, unstable dynamics [24, 25, 26, 27, 28, 29, 30, 31], thus presenting a failure in keeping a safety-margin from instability. Multiple computational models suggest that entering a seizure could correspond to a change in the brain's dynamical state, via a bifurcation or critical transition [32, 31, 33]. Furthermore, experimental evidence suggests that neural activity during epileptic seizures and epileptiform activity deviates from healthy activity in indicators of criticality [26, 27], and neural activity during seizure termination has shown signatures of a critical transition across different recording levels [34]. Recently, long-term changes in signatures of critical slowing down, together with epileptiform discharges, were shown to be a reliable indicator for seizure risk [35]. In contrast to these findings, recent studies on human iEEG did not find consistent warning signals of a critical transition prior to seizures [36, 37], raising the question of whether, and under which conditions, the framework of criticality can capture the mechanisms that underlie seizure generation.

2. Choice to time window:

The authors limit their investigation to a few minutes before seizure onset and leave out the seconds right before seizure onset (due to understandable methodological constraints). However, it be carefully discussed already in the introduction of the paper; conclusions made from the limited time window observations should be more cautious and take references (1) and (3) into account (see also next point for another concern that the authors do not discuss in their findings).

Thank you for pointing this out, we agree that a careful discussion of the time window is important. We extended our discussion on the time window, taking into account the studies by Chang et al. and Maturana et al. For a more detailed response, see Reviewer 2, question (10).

3. Lack of consideration of noise effects:

The authors do not consider the role of noise in the interpretation of their findings. Broadly speaking, critical slowing down can only be observed under moderate noise levels; if noise is too large, then it will cause a transition to the new state (crossing the bifurcation point) even if the bifurcation point is still relatively far away. The Maturana et al. 2020 manuscript includes a more detailed discussion of these matters as well as the more theoretical accounts on critical transitions provided in Kuehn 2011 (https://arxiv.org/abs/1101.2899). The authors may want to include a critical discussion of these noise aspects. For example, it may be conceivable that critical slowing down (i.e. the distance to criticality) is shaped on much longer timescales, such as days (Maturana et al., 2020) and the eventual seizure onset is then primarily driven by noise that pushes the system into the seizure state once it is sufficiently close to criticality.

Both observational as well as dynamical noise (or variability) may indeed play a decisive role. We had tried to point out the idea that precisely the dynamical noise drives the system across the bifurcation or

transition in an unpredicive manner. Moreover, the slow drifts over hours or days you point out above may play a further role. We now put that aspect in a broader context than before, and added it to our discussion. The respective part of the paragraph reads:
[...] Or the transition could occur on a much slower time scale, such that changes during the last 10 minutes are too small to be detectable. Once the system is sufficiently close to criticality, it could spontaneously be driven into a seizure by noise. This hypothesis is supported by a recent study that found signatures of critical slowing on a timescale of hours to days before seizure onset in human iEEG [35].
Furthermore, as suggested by Reviewer 2, we now also analyzed the variability in the branching parameter when approaching seizure onset, as increasing fluctuations in the branching parameter could be indicative for noise-driven seizure onset. However, we find no significant change during the last 10 minutes. Thus according to our data, both, systematic trends and changing variability in m, are not shaped on a timescale of several minutes.

4. A more balanced overview of the literature:
There a several statements in the manuscript that do not take into account other studies and the manuscript could benefit from being a bit more cautious in its conclusions by also giving a more broad, balanced account of the literature. Some statements may actually be a bit ill-fated or potentially even wrong in light of the literature that is not scanned appropriately and considered at present. Some examples (author statements set in italic):

Thank you for the critical reading and the detailed feedback. We would like to address the criticized statements individually:

Such inherent unpredictability is characteristic for critical systems, and may provide an explanation why reliable approaches for seizure prediction have not been established to date [33,34].
One may equally argue the opposite: the fact that seizure prediction is at all possible (Cook et al., 2013; and others) indicates that there are systematic changes in the neuronal signals that are being picked up by prediction algorithms (which could, in principle, be related to critical slowing down). I suggest to state this a bit more cautiously.

We agree that reliable seizure prediction may become possible in future. To date, however, it remains inherently difficult. The clinical trial of a seizure prediction device (Cook et al., 2013) showed a better-than-chance warning performance in nine of 14 participating patients. In a subsequent Kaggle competition on data from humans and canines, in which 10-min recordings had to be classified into preictal or interictal, several competing algorithms achieved significantly better-than-chance-performance (Brinkmann et al., 2016). While these results are promising, reliable seizure prediction requires more than better-than-chance and we do not consider the problem of seizure prediction solved to date.
Indeed, if seizure onset was preceded by a transition towards criticality, it could be predictable, as we hypothesized in scenario B (Fig. 1c in main text). If, on the other hand, the dynamics are very close to instability for extended periods of time (e.g. due to long-term fluctuations or a generally different dynamical state), the transition can be driven by noise and thus not be predictable (hypothetical scenario A, Fig. 1b in main text). Nevertheless, we generally rewrote the respective paragraph in the introduction, adding a broader perspective on previous studies, and leaving out the discussed sentence (see response to point (1)).

In the first scenario, one expects the seizure onset zone (SOZ) to generally operate closer to instability, and thus a clear difference between the SOZ and the same brain area in the conta- lateral, nonfocal hemisphere (nSOZ) (Fig. 1b).
This hypothesis, as stated, ignores a large body of research indicating epilepsy to be a network disease, where regions far from the SOZ may be more indicative of an upcoming seizure.

Thank you for pointing this out, our explanation might indeed have been too short here. The actual

idea is that a brain area in which seizures emerge may operate closer to instability than the same area in a *healthy* brain. As spiking data is not accessible from healthy humans, we use the contra-lateral hemisphere as an approximation for healthy (which can be justified by the fact that most patients were fully or partially seizure-free after resection of this area). We now made this more clear both in the introduction and the discussion:
Introduction: In the first scenario, one expects brain areas, in which seizures emerge, to generally operate closer to instability than the same areas in a healthy human brain, so that small fluctuations can push the system into a seizure (Fig. 1b).[...]
To assess whether epileptic areas generally operate closer to instability, we compared the medial temporal lobe (MTL) containing the epileptic focus to the same area in the contra-lateral hemisphere, as an approximation of a healthy control.
Discussion: We found no evidence that the hemisphere, in which seizures emerge, generally operates closer to instability. Although there were systematic differences between focal and contra-lateral hemisphere in some patients, these differences were not consistent across patients. One potential explanation is that the epileptic focus might only consist in a small, localized population of neurons that was not always captured by the recording electrodes. This hypothesis is supported by evidence from epileptic seizures indicating a core territory of recruited neurons that is surrounded by a so-called ictal penumbra [51]. On the other hand, a variety of studies suggest that epilepsy is a network disease, and pre-seizure changes have been observed in neurons far away from the epileptic focus [29, 49]. Thus, it is conceivable that the entire brain operates in a different dynamical regime, which would explain the lack of a systematic difference between the hemispheres. Investigating this hypothesis would ideally require single neuron data from healthy humans, which are, however, practically not accessible with existing recording techniques.

To quantify m, we made use of a novel, unbiased estimator that only requires knowing the number of spikes sampled from a small set of neurons to return a reliable estimate of the branching parameter m [36]. Importantly, the estimator is invariant under subsampling [37], thus it can infer the propagation of activity in a network even when recording only a small fraction of all neurons [12,38].
This estimator is essentially a fit parameter to the autocorrelation function of neuronal activity. The authors may consider citing other studies that have used the spiking autocorrelation function to infer distance to criticality (e.g. Bellay et al., 2015 Irregular spiking of pyramidal neurons organizes as scale-invariant neuronal avalanches in the awake state; Meisel et al., 2017 The interplay between long- and short-range temporal correlations shapes cortex dynamics across vigilance states).
Indeed, the estimation of the branching parameter m is based on the decay time of the autocorrelation function of neuronal activity. We now highlight this aspect more, e.g. in the introduction:
More generally, an increasing branching parameter indicates more temporally correlated neural activity [12] and is therefore a signature of *critical slowing*, which is expected when a system approaches a critical transition [25].
Also, we added the study by Meisel et al., 2017, as it provides important evidence for a dependence of the distance to criticality on the vigilance state:
The autocorrelation structure of neuronal activity, and therefore the branching parameter m, can change depending on vigilance state and circadian rhythm [45, 35, 37, 46].

For longer putative pre-seizure periods (30 minutes to 4 hours), signatures of 'critical slowing down', which are associated with transitions to supercriticality, have not been found either [32], which is consistent with our results.
Please consider references 1 and 3 above, which claim the opposite of what you write, and kindly balance your statement.
We agree that this statement must be adjusted considering the findings by Maturana et al., 2020. Please understand that the study by Maturana et al., 2020 had not been not published at the time of submis-

sion. The study by Chang et al., 2018 did indeed also look at pre-ictal changes in humans, however, they did not find a consistent trend across patients: some patients showed an increase (4/12), others a decrease (4/12), the rest showed no significant trend (4/12) in the measured autocorrelation coefficient. We now added both studies to the discussion (see Reviewer 2, question (10)).

Thus for seizure prediction as early warning, which would be important for patients to seek a safe environment before a seizure starts, the framework of criticality has not proven useful yet.
Please consider reading Maturana et al., 2020, where the incorporation of critical slowing down into seizure forecasting algorithms has provided the best seizure forecasting algorithm to date. The above sentence is thus simply incorrect.

We agree that our statement must be corrected in the light of the recent results presented by Maturana et al.. The sentence now reads as follows:

Thus it remains to be investigated precisely, under which conditions (e.g. types of epilepsy and types of recordings) the framework of criticality can serve to predict an impending epileptic seizure.

In line with this idea, the study that found signatures of critical slowing during seizure termination found these signatures only on the more coarse recording levels (EEG, ECoG, LFP), not in multi unit activity [30]. Therefore, the sparsely recorded single unit activity may not be the ideal type of signal to identify seizure onset dynamics.
As mentioned above, this important concern about the choice of methods should be emphasized at the beginning already as as it is essential to interpretation of all results.

See response to point (1).

In summary, we found no evidence for epilepsy to involve a transition towards supercritical dynamics
This statement, in its generality, may be misleading since the authors never present results from an analysis of ictal data. Ictal dynamics may or may not be supercritical, it is simply not being studied Thein the present manuscript.

To avoid misinterpretation, we now formulated the sentence in a more detailed way and added the previously discussed aspects to the conclusion:

In summary, we found no evidence for a transition towards supercritical dynamics in pre-seizure single neuron activity of human cortex. This finding is in line with previous studies finding no warning signals of a critical transition prior to epileptic seizures [37, 36], but stands in contrast with a recent study demonstrating the effectiveness of signatures of critical slowing for seizure prediction [35]. It remains to be investigated precisely whether single-unit recordings, despite providing the most direct information on neuronal activity, cannot resolve pre-seizure changes in criticality. Furthermore, it is conceivable that seizure activity proper is indeed supercritical, with dynamics crossing the critical thresholds only seconds before seizure onset. Alternatively, seizure generation might be a qualitatively different process that cannot be captured by a linear stability parameter m.

5) A more comprehensive analysis of the autocorrelation estimator
The authors report no systematic change in the autocorrelation structure (assessed by their estimator) across cortical regions or across time. One may thus ask whether their estimator can confirm other findings reported in the literature in order to determine whether the indicator is robust/valid: - For example: does the indicator show a change during seizures? It may be argued that seizure dynamics is very different from interictal dynamics, leading often to a complete or partial functional loss of the affected cortical region. Is the estimator able to capture such a change? Why did the authors not report on findings during seizures? This would be very interesting and seems like a missed opportunity. I understand that findings may vary widely and there may be no coherent picture emerging. Nevertheless, the manuscript might benefit strongly

by including such an analysis, even if it means reporting non-coherent findings that vary across seizures and patients. Given the brevity of the study and the importance to assess what the estimator picks up during a seizure, I think an extension of the analysis to include ictal data would be crucial. - Similarly, does the estimator show a change at the offset of the seizure, such as reported in Kramer et al., 2012? - Does the estimator confirm a hierarchy of temporal scales, as reported e.g. in Murray et al., 2014 (also cited by the authors)? This work uses, very similarly, the autocorrelation function and has reported an increase in timescales with cortical hierarchy. The authors already provide subanalyses for different regions; could this be extended to compare regions with low and high levels of hierarchy? I think these questions are important to better understand what the authors' indicator actually measures. If it measures the same values at all times and cannot confirm findings from other studies in the past, then one may question these earlier studies or, alternatively, be cautious about validity of the indicator used in the present study.

The MR estimator has been evaluated in great detail in its original publication (Wilting & Priesemann, 2018). Among others, the estimates of the distance to criticality were verified for the branching process model, a branching network, and the Bak-Tang-Wiesenfeld model. Furthermore, as you correctly pointed out above, the MR estimator is based on measuring the autocorrelation time of spiking activity. We are currently applying it to data from a full hemisphere of macaque monkey and are able to confirm the results by Murray et al., 2014 (work in preparation). Thus we can confidentially state that the MR estimator captures differences in the autororrelation structure.

In humans, we only have access to data from the medial temporal lobe (MTL). Across subregions of MTL, we do not find strong differences – which is not surprising, as the entire MTL is located high in the hierarchy. However, we do have first evidence from the human data that there are differences between sleep stages (work in preparation).

We agree that it would be highly interesting to analyze the branching parameter *during* seizures. Unfortunately, the acquisition of single neuron recordings during a seizure is technically extremely difficult due to increased movement artifacts and failure of spike sorting (see also e.g. Merricks et al., Brain 2015). Therefore, although we would like to investigate this aspect, we are limited to the stable interictal and preictal recordings.

6. Exclusion criteria of MR estimation:

Could the authors please elaborate more on the impact of the the second exclusion criterion? As is well known, the autocorrelation function will decay geometrically at criticality. It can be argued that a power-law decay may look more like a linear decay than an exponential decay, particularly on short time lags. The linear fit may thus potentially be the better fit at criticality. Thus, requiring that the autocorrelation function must be better fit by an exponential than by a linear fit may impose a systematic bias towards subcriticality (where it decays exponentially)? Could the authors please elaborate?

This is indeed an important methodological question. The exclusion criterion captures two situations (1) if the ACF is basically just noise (no visible decay), which is expected for Poisson-like activity, and (2) if the decay of the ACF appears linear, which can be caused by non-stationarities in the time series (see Supplementary of Wilting & Priesemann 2018).

Indeed, at criticality, the ACF is expected to show a power-law decay and we agree that for short time lags, this can be mistaken as a linear decay (more specifically, the decrease of the ACF would be slow, but with values clearly above zero). The underlying, very general issue is that criticality comes with diverging autocorrelation times $\tau \to \infty$, but recording time is limited. Estimating m in this regime would therefore correspond to fitting autocorrelation times that are much longer than recording length.

We did discuss about whether to include or to exclude those recordings, and we are open to a debate on that question. For our specific dataset, we consider the exclusion the more methodologically solid approach, because the *inclusion* would come with the methodological weaknesses of (i) including recordings that are subject to other effects, such as non-stationarities or high noise, and (ii) fitting autocorrelation times that are longer than recording length, which is not reliable in the presence of noise. Furthermore, we find that the number of recordings that is excluded due to linear decay is very small. In

the analysis of the full MTL, for instance, there are only 3 out of 176 recordings[1]. Unlike the expectation for a critical system, the excluded ACFs have absolute values close to zero and a high signal-to-noise ratio. Fig. 6 shows the corresponding ACFs as well as the activity time series. In our view, it is more sound to exclude these estimates, because (i) the estimated autocorrelation times represent outliers compared to the overall distribution of estimates and (ii) the signal-to-noise ratio for these recordings is quite high, so that the fit has a high uncertainty.
In total, this leads us to keep the exclusion criterion, but to add a discussion of this aspect to the methods to make the readers explicitly aware that the exclusion criterion can lead to an exclusion of critical recordings.
Note that at critiality, the intrinsic timescale $\hat{\tau}$ is expected to diverge. The autocorrelation function of dynamics very close-to-critical on a limited number of time steps can therefore be mistaken with a linear decay. We nevertheless implemented the second exclusion criterion, as it captured erroneous fits due to non-stationarities in the recording or high signal-to-noise ratios in our dataset. In general, however, the use of the second criterion must be assessed carefully for any given dataset, to ensure that it does not introduce a bias towards subcritical dynamics.

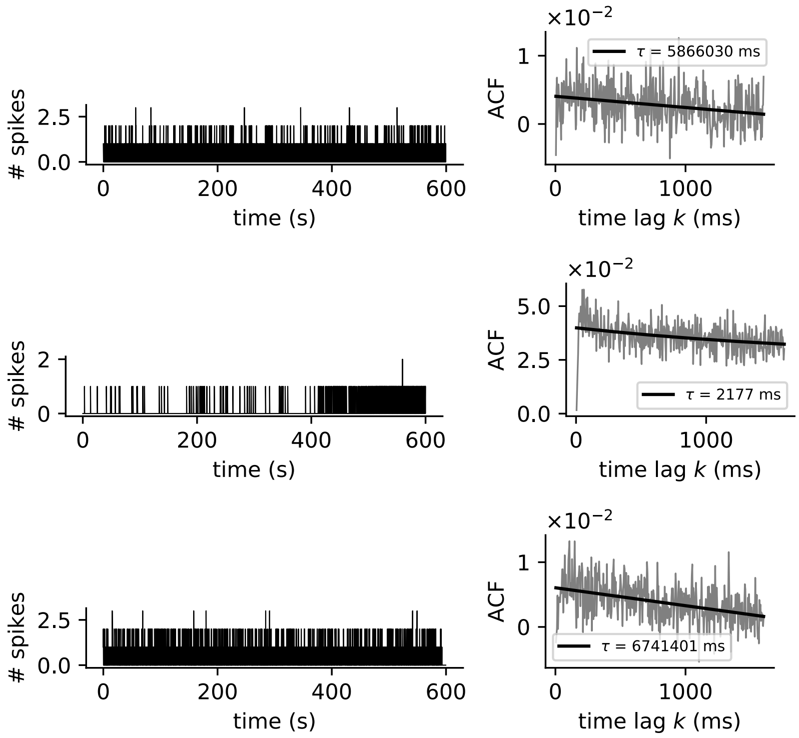

Fig 6. Recordings excluded based on the linear exclusion criterion. The autocorrelation functions (ACF) of the first and last recording show a high signal-to-noise ratio and the estimated intrinsic timescales τ are unusually long compared to the remaining recordings. In the second recording, the population firing rate increases over time. Based on visual inspection, the fit to the ACF does not capture the real decay, but is dominated by the first few datapoints.

7. The authors should please confirm and state explicitly that, when comparing different cortical regions in one patient, data was collected from the same time intervals, as it is known that the autocorrelation structure of neural signals is also strongly influenced by circadian rhythms and AED action (e.g. ref. 47).

[1]These are the recordings that were rejected by the linear exclusion criterion, but that would not have been rejected by comparison with a *flat* linear function. Comparison is always performed taking into account the different numbers of parameters.

The different influences on the autocorrelation structure of neural signals are indeed an important point, thank you for highlighting it. The patient-wise results in Supp. Figure S2 do indeed show pairs from the same time intervals. The statistical summary (Figure 1), however, shows the results across recordings and patients and therefore does not explicitly account for time intervals. In this overall summary, we combine all data of the respective region to find out whether there is an overall measurable effect. To verify that circadian rhythms and AED action do not overshadow an effect, we now also added the same figure, but only including paired data, to the supplementary (see Fig. 7).
The autocorrelation structure of neuronal activity, and therefore the branching parameter m, can change depending on vigilance state and circadian rhythm [45, 35, 37, 46]. Furthermore, anti-epileptic drugs modulate cortical excitability and were shown to affect the branching parameter [47, 48]. To make sure that temporal differences do not overshadow differences between the focal and the contra-lateral hemisphere, or wrongfully give rise to apparent differences, we performed the same analyses using only *paired* recordings (recordings obtained from the same patients in the same time intervals). Consistent with the results shown in Fig. 1d,f, we found a significant difference between the focal and the contra-lateral hemisphere in hippocampus, and no significant differences in the remaining subregions and the full MTL (Fig. S3).

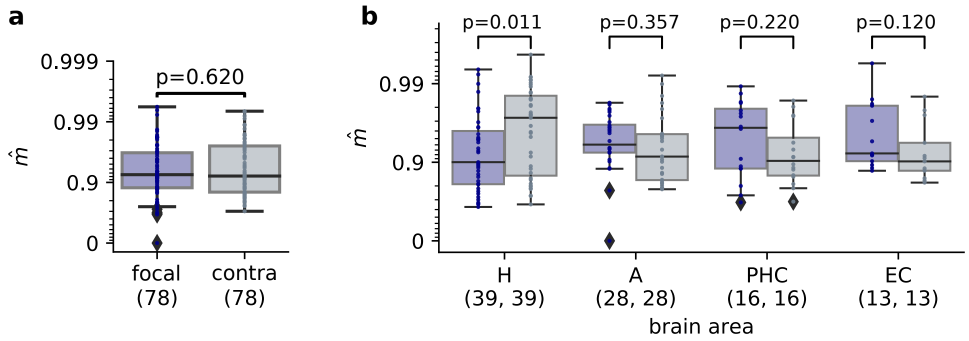

Fig 7. (Copy of Fig. S3 in the supplementary of the manuscript). Comparison of focal versus contra-lateral hemisphere using only *paired* data, i.e. pairs of data that were recorded simultaneously in the focal and the contra-lateral hemisphere. **a** The branching parameter $\hat{m}$ across recordings and patients shows no significant difference between focal and contra-lateral hemisphere. **b** Comparison of the branching parameter $\hat{m}$ in focal and contra-lateral hemisphere for different subregions of the MTL, (hippocampus (H), amygdala (A), parahippocampal cortex (PHC) and entorhinal cortex (EC)). The hippocampus shows a just significant difference between focal and contra-lateral hemisphere, with a smaller m in the focal hemisphere. The other subregions show no significant difference ($p < 0.05/4$ required after Bonferroni correction).

Response to reviewer 4

Reviewer 4: *This manuscript examines the role of dynamical stability/instability in epilepsy, in particular by testing the hypothesis that epiletic seizures "reflect a transition to supercritical, unstable dynamics" in either of two disctinct scenarios: (a) epileptogenic areas already operate closer to instability (than nonepileptogenic areas in the same patient) during ongoing interictal periods, or (b) epileptogenic areas approach instability gradually during a preictal period leading to the seizure onset.*
A branching process is used to assess stability by modeling the time evolution of population activity (number of action potentials in given time bins) computed from recorded single-units in both brain hemispheres ("focal" and "non-focal")20 patients. The branching parameter m is estimated from data. When $m < 1$, the system is subcritical (stable), critical for $m = 1$, and supercritical for $m > 1$. To examine (a) and (b) above, fixed and time varying m are consider.
Estimated parameters reveal that epileptogenic areas show subcritical regimes throughout the examined interictal periods, excluding the two above hypothesized scenarios. Overall, it appears that neuronal activity in epileptogenic areas during interictal and near seizure periods are no different from near criticality, but still subcritical, regimes previously identified in various other normal or healthy state conditions. My main concerns relate to the nature of this manuscript's contribution and its suitability to PLoSCB, and other more technical issues described in more detailed below.
In conclusion, I think a substantially revised and extended version of the manuscript to address the above issues would be more indicated to a specialized journal in clinical neuroscience or epilepsy.

We thank you for the careful assessment of our manuscript. Before we address the points one by one, let us start with outlining the general motivation of this study, and the motivation to publish these "negative" results in the present form.
We started off with two clear hypotheses about how changes in the distance to criticality might be related to seizure dynamics (Figs. 1b,c in the manuscript). With the intracranial spike recordings, we could test these hypotheses. Unfortunately, we found no evidence for either of them. Faced with these negative results, in practice one can then either revise the hypothesis, investigate a different set of parameters or observables, e.g. by switching from a branching process instability to a framework of metastability or oscillation, and eventually one might find a hypothesis for which the data provide significant results. However, this approach can easily lead to false-positive reports. Therefore, we decided to stay with the original hypotheses, which had been formulated in all detail in a grant proposal, and then report the negative results. We believe that this is scientifically the most rigorous path to follow, though not necessarily the most rewarding. As a consequence, we have to report negative results on this specific hypotheses. However, it does not exclude that other perspectives on critical phenomena might still play a role in seizure dynamics.
We added this clarification to the discussion, and we hope that it increases the acceptance of publishing negative results. This obviously does not contradict that we should put all effort into investigating the hypothesis with all care. We thank you, and the other reviewers, for your helpful input on that.

(1) The manuscript does not make an important methodological or modeling contribution. Branching process and the notion of critical branching have both been examined by many groups (including the authors) in previous decades and in various contexts, including epileptic seizures. Furthermore, the estimator for the branching parameter used here has been introduced and applied to neural data by the same group in a previous publication (Nature Comm, 2018).
The modeling contribution of our work does indeed not lie in the branching process or the Multistep-Regression estimator. The core aim also is not to develop a novel model, but to revisit a popular hypothesis in the field. Hence, the contribution is about the dynamics of epilepsy and seizure generation. The hypothesis that seizures might be related to a critical transition has been brought forward multiple times, but evidence is limited. With our work, we connect recently developed tools to assess criticality

with single-neuron data from epilepsy patients. This is indeed novel, as existing studies on criticality and epilepsy were always restricted to more coarse recordings (EEG, iEEG, LFP, MEG) and thus coarse approximations of the branching parameter, or other signatures of criticality. We are not aware of any other study that used spiking activity to investigate critical slowing in epilepsy.
Moreover, the time-resolved analysis using sliding windows is in fact methodologically novel. We did not stress this aspect of novel analysis, but in the background had carefully checked and validated the choice of the analysis parameters (see SI).
Therefore, our work provides not only an assessment of a popular hypothesis, but also an important basis for future studies that aim at modeling or analyzing the dynamics of seizure generation or the dynamics of criticality (and deviations thereof) in spiking data.

(2) Contrast with other related results: Despite Wilkat et al. (2019)[32] and Milanowski and Suffczynski (2016)[31], several other groups have argued (based on both theoretical and experimental results) that focal seizures arise as brain dynamics loose stability when approaching particular types of bifurcations (at seizure onset). For recent work, see for example Maturana et al. (Nature Comm, 2020) providing evidence for critical slowing prior to seizure onset based on intracranial EEG, or recent work from the Jirsa group. (This reviewer is not a co-author in those articles.) The dynamics in those cases are more complex than branching processes, especially with bifurcations into oscillations, etc. Thus, the issue seems to remain pretty open, and so the negative results presented in this study require a much more elaborate and detailed analyses, given potential issues including amount and type of data, model mismatch, etc.
We agree that more future research is needed to get a full understanding of whether, and under which conditions, the framework of criticality can serve to model the dynamics of epilepsy. Nevertheless, our work brings an important piece of evidence to the field, by suggesting that single-neuron activity close to the seizure onset zone is not generally shifted towards supercritical dynamics and does not systematically drift towards supercriticality during the last 10min before seizure onset. Future work can build on these new findings, until a full understanding is achieved.
Furthermore, regarding the type of bifurcation, we don't consider the branching process model a limiting factor. Similar to the metrics analyzed by Marutana et al., the branching parameter is a signature of critical slowing. Thus, although we specifically consider the branching process model, we are analyzing a signature of critical slowing. Such critical slowing is to be expected before any second order phase transition, including those proposed by Marutana et al.
We specifically focused on the notion of critical branching because our and recent studies (e.g. Wilting & Priesemann 2019) suggest that the statistics of cortical network activity are well approximated by a slightly subcritical branching model, which motivates the hypothesis that seizures might be related to a tipping over to $m > 1$. Nevertheless, we totally agree that other types of bifurcations are also possible and we now added a discussion alternative types of bifurcations in the discussion of our paper.
In the literature, a variety of alternative mechanisms for seizure generation have been proposed. While we focused on criticality using the framework of a branching process, several other models describe seizure generation by a critical transition (bifurcation), but with different order and control parameters [59, 31, 32, 35]. While the specific dynamics of these models differ, they all involve a deterministic change in a control parameter that could, in principle, be detectable. In fact, it is expected that all mechanisms, in which the transition to seizures involves a second order phase transition, will show signatures of critical slowing [25]. As the branching parameter m is an indicator for critical slowing, it should increase if cortical dynamics approach a second order phase transition, independent of the specific underlying path. Thus, our analysis is not restricted to a branching process model, but measures the proximity of cortical dynamics to a second order phase transition.
On the other hand, there are models that do not describe seizures via bifurcations, but with multi-stable models, in which seizure onset is not driven by deterministic parameter changes, but by random fluctuations [60, 32]. In these models, seizures would be inherently unpredictable, as random fluctuations in the dynamics drive the system into the seizure state. Such a model could, in principle, explain our negative finding. However, there is accumulating evidence that seizures are, to a certain extent, predictable, which indicates that seizures are likely not only driven by noise [35, 61, 62].

Finally, a combination of mechanisms is possible: deterministic changes in the stability could lead to an increased seizure risk, accompanied by random fluctuations that drive the system into the seizure [63]. In this case, it is conceivable that the deterministic changes happened on a timescale longer than detectable in our 10-minute analysis, and entering the seizure was then driven by random fluctuations.

(3) The authors acknowledge their negative findings could have resulted from not including data very close to seizure onsets. As acknowledged, the negative results could be explained by the possibility that loss of stability might occur in a much faster way and during a much shorter period culminating with the seizure onset.

We agree that this is a limitation of the temporal analysis and we therefore further expanded our discussion on the time window in the manuscript. However, most experimental approaches have some limitation in their resolution and we do not consider this a reason for not publishing the results that are accessible (of course with adequate discussion). In fact, for application in seizure prediction, the analyzed timescale of minutes would be of particular interest.

For a more detailed response and the expanded discussion in the manuscript, see Reviewer 2, question (10).

(4) Also, a critical limitation of this manuscript is that beyond the negative results, no insights or alternative models are provided to shed new light on how brains transition into seizures. Given the results, it seems the case that brains do not transition into seizures via bifurcations (loss of stability). Which alternative generic dynamics (multi/meta-stability, or something else) and models? How data and statistical properties, etc, would differ in these different cases?

Thank you for pointing out this limitation. We initially focused strongly on compactness – however we do agree that our manuscript does indeed benefit from a broader perspective on alternative models. We therefore added a paragraph in the discussion (see question (2)).

(5) While my assessement of the manuscript is based on the above, I would also like to comment on the potential issue of model mistmatch. For example, as the authors know, the equality $= m$ for the (macroscopic) branching parameter in the conditional expectation (Eq. 1) follows from the random variables $Y_{t,i}$ being i.i.d. over neurons (i.e. sufficient homogeneity in the properties and independent activity of neurons in a network). This can be justified (via mean-field approaches, etc) for some models of large neuronal networks, but is far from being a feature of general networks or known to be case for actual neuronal networks of interest. Yes, all models are wrong, but some are usefull. The authors and others have provided some evidence branching processes capture several statistical properties of the population activity in various previous studies, although I haven't seen analysis looking at more detailed aspects such as how well probability of full sample paths are captured. Should a more refined definition of population (depending on neuronal subtypes and general cortical/neocortical network structure) matter? In some cases, it all might "average out" and not matter, but that's not clear. Furthermore, the invariance to subsampling of the branching parameter estimator should depend on how well the branching process model captures the dynamics of the target neuronal networks.

This is indeed an interesting discussion. The branching process (and the branching parameter) is certainly a simplistic model. However, as you pointed out, there are strong statistical similarities of cortical activity and the branching process as shown e.g. by Wilting et al. (2018).

Furthermore, as discussed above, our findings do not depend on the branching process model: Leaving the branching process model aside, the branching parameter essentially measures the autororrelation time of neuronal activity. Independent of the branching process model, a high autocorrelation time (corresponding to a high m), is a signature of critical slowing. Thus, although the branching process is a widely used model in the field and provides an intuitive understanding, our results do not depend on this model. Even without this model, we find no evidence for critical slowing in single neuron activity of epilepsy patients.

References

[1] Shew WL, Plenz D. The functional benefits of criticality in the cortex. The neuroscientist. 2013;19(1):88–100.

[2] Haldeman C, Beggs JM. Critical Branching Captures Activity in Living Neural Networks and Maximizes the Number of Metastable States. Physical Review Letters. 2005;94(5):058101. doi:10.1103/PhysRevLett.94.058101.

[3] Kinouchi O, Copelli M. Optimal dynamical range of excitable networks at criticality. Nature physics. 2006;2(5):348.

[4] Bertschinger N, Natschläger T. Real-time computation at the edge of chaos in recurrent neural networks. Neural computation. 2004;16(7):1413–1436.

[5] Priesemann V, Wibral M, Valderrama M, Pröpper R, Le Van Quyen M, Geisel T, et al. Spike avalanches in vivo suggest a driven, slightly subcritical brain state. Frontiers in Systems Neuroscience. 2014;8. doi:10.3389/fnsys.2014.00108.

[6] Wilting J, Dehning J, Pinheiro Neto J, Rudelt L, Wibral M, Zierenberg J, et al. Operating in a Reverberating Regime Enables Rapid Tuning of Network States to Task Requirements. Frontiers in Systems Neuroscience. 2018;12. doi:10.3389/fnsys.2018.00055.

[7] Beggs JM, Plenz D. Neuronal avalanches in neocortical circuits. The Journal of Neuroscience: The Official Journal of the Society for Neuroscience. 2003;23(35):11167–11177.

[8] Friedman N, Ito S, Brinkman BA, Shimono M, DeVille RL, Dahmen KA, et al. Universal critical dynamics in high resolution neuronal avalanche data. Physical review letters. 2012;108(20):208102.

[9] Zierenberg J, Wilting J, Priesemann V. Homeostatic Plasticity and External Input Shape Neural Network Dynamics. Physical Review X. 2018;8(3):031018. doi:10.1103/PhysRevX.8.031018.

[10] Neto JP, Spitzner FP, Priesemann V. A unified picture of neuronal avalanches arises from the understanding of sampling effects. arXiv preprint arXiv:191009984. 2019;.

[11] Wilting J, Priesemann V. 25 years of criticality in neuroscience—established results, open controversies, novel concepts. Current opinion in neurobiology. 2019;58:105–111.

[12] Wilting J, Priesemann V. Between Perfectly Critical and Fully Irregular: A Reverberating Model Captures and Predicts Cortical Spike Propagation. Cerebral Cortex (New York, NY: 1991). 2019;29(6):2759–2770. doi:10.1093/cercor/bhz049.

[13] Dahmen D, Grün S, Diesmann M, Helias M. Second type of criticality in the brain uncovers rich multiple-neuron dynamics. Proceedings of the National Academy of Sciences. 2019;116(26):13051–13060.

[14] Munoz MA. Colloquium: Criticality and dynamical scaling in living systems. Reviews of Modern Physics. 2018;90(3):031001.

[15] Hahn G, Petermann T, Havenith MN, Yu S, Singer W, Plenz D, et al. Neuronal avalanches in spontaneous activity in vivo. Journal of neurophysiology. 2010;104(6):3312–3322.

[16] Cocchi L, Gollo LL, Zalesky A, Breakspear M. Criticality in the brain: A synthesis of neurobiology, models and cognition. Progress in neurobiology. 2017;158:132–152.

[17] Aburn MJ, Holmes C, Roberts JA, Boonstra TW, Breakspear M. Critical fluctuations in cortical models near instability. Frontiers in physiology. 2012;3:331.

[18] Ribeiro TL, Copelli M, Caixeta F, Belchior H, Chialvo DR, Nicolelis MA, et al. Spike avalanches exhibit universal dynamics across the sleep-wake cycle. PloS one. 2010;5(11):e14129.

[19] Ribeiro TL, Ribeiro S, Belchior H, Caixeta F, Copelli M. Undersampled critical branching processes on small-world and random networks fail to reproduce the statistics of spike avalanches. PloS one. 2014;9(4):e94992.

[20] Fagerholm ED, Scott G, Shew WL, Song C, Leech R, Knöpfel T, et al. Cortical entropy, mutual information and scale-free dynamics in waking mice. Cerebral cortex. 2016;26(10):3945–3952.

[21] Ponce-Alvarez A, Jouary A, Privat M, Deco G, Sumbre G. Whole-brain neuronal activity displays crackling noise dynamics. Neuron. 2018;100(6):1446–1459.

[22] Ma Z, Turrigiano GG, Wessel R, Hengen KB. Cortical circuit dynamics are homeostatically tuned to criticality in vivo. Neuron. 2019;104(4):655–664.

[23] Marshel JH, Kim YS, Machado TA, Quirin S, Benson B, Kadmon J, et al. Cortical layer–specific critical dynamics triggering perception. Science. 2019;365(6453):eaaw5202.

[24] Hsu D, Chen W, Hsu M, Beggs JM. An open hypothesis: is epilepsy learned, and can it be unlearned? Epilepsy & behavior : E&B. 2008;13(3):511–522. doi:10.1016/j.yebeh.2008.05.007.

[25] Scheffer M, Bascompte J, Brock WA, Brovkin V, Carpenter SR, Dakos V, et al. Early-warning signals for critical transitions. Nature. 2009;461(7260):53–59. doi:10.1038/nature08227.

[26] Meisel C, Storch A, Hallmeyer-Elgner S, Bullmore E, Gross T. Failure of Adaptive Self-Organized Criticality during Epileptic Seizure Attacks. PLOS Computational Biology. 2012;8(1):e1002312. doi:10.1371/journal.pcbi.1002312.

[27] Arviv O, Medvedovsky M, Sheintuch L, Goldstein A, Shriki O. Deviations from Critical Dynamics in Interictal Epileptiform Activity. Journal of Neuroscience. 2016;36(48):12276–12292. doi:10.1523/JNEUROSCI.0809-16.2016.

[28] Hobbs JP, Smith JL, Beggs JM. Aberrant Neuronal Avalanches in Cortical Tissue Removed From Juvenile Epilepsy Patients. Journal of Clinical Neurophysiology. 2010;27(6):380. doi:10.1097/WNP.0b013e3181fdf8d3.

[29] Yan J, Wang Y, Ouyang G, Yu T, Li Y, Sik A, et al. Analysis of electrocorticogram in epilepsy patients in terms of criticality. Nonlinear Dynamics. 2016;83(4):1909–1917. doi:10.1007/s11071-015-2455-9.

[30] Chang WC, Kudlacek J, Hlinka J, Chvojka J, Hadrava M, Kumpost V, et al. Loss of neuronal network resilience precedes seizures and determines the ictogenic nature of interictal synaptic perturbations. Nature neuroscience. 2018;21(12):1742–1752.

[31] Meisel C, Kuehn C. Scaling effects and spatio-temporal multilevel dynamics in epileptic seizures. PLoS One. 2012;7(2):e30371.

[32] Da Silva FL, Blanes W, Kalitzin SN, Parra J, Suffczynski P, Velis DN. Epilepsies as dynamical diseases of brain systems: basic models of the transition between normal and epileptic activity. Epilepsia. 2003;44:72–83.

[33] Jirsa VK, Stacey WC, Quilichini PP, Ivanov AI, Bernard C. On the nature of seizure dynamics. Brain. 2014;137(8):2210–2230.

[34] Kramer MA, Cash SS. Epilepsy as a Disorder of Cortical Network Organization , Epilepsy as a Disorder of Cortical Network Organization. The Neuroscientist. 2012;18(4):360–372. doi:10.1177/1073858411422754.

[35] Maturana MI, Meisel C, Dell K, Karoly PJ, D'Souza W, Grayden DB, et al. Critical slowing down as a biomarker for seizure susceptibility. Nature Communications. 2020;11(1):1–12.

[36] Milanowski P, Suffczynski P. Seizures Start without Common Signatures of Critical Transition. International Journal of Neural Systems. 2016;26(08):1650053. doi:10.1142/S0129065716500532.

[37] Wilkat T, Rings T, Lehnertz K. No evidence for critical slowing down prior to human epileptic seizures. Chaos: An Interdisciplinary Journal of Nonlinear Science. 2019;29(9):091104. doi:10.1063/1.5122759.

[38] Harris TE. The Theory of Branching Processes. Grundlehren der mathematischen Wissenschaften. Berlin Heidelberg: Springer-Verlag; 1963. Available from: https://www.springer.com/de/book/9783642518683.

[39] Wilting J, Priesemann V. Inferring collective dynamical states from widely unobserved systems. Nature Communications. 2018;9(1):2325. doi:10.1038/s41467-018-04725-4.

[40] Hsu D, Beggs JM. Neuronal avalanches and criticality: A dynamical model for homeostasis. Neurocomputing. 2006;69(10):1134–1136. doi:10.1016/j.neucom.2005.12.060.

[41] Badawy R, Freestone D, Lai A, Cook M. Epilepsy: ever-changing states of cortical excitability. Neuroscience. 2012;222:89–99.

[42] Priesemann V, Munk MH, Wibral M. Subsampling effects in neuronal avalanche distributions recorded in vivo. BMC neuroscience. 2009;10(1):40.

[43] Spitzner F, Dehning J, Wilting J, Hagemann A, Neto J, Zierenberg J, et al. MR. Estimator, a toolbox to determine intrinsic timescales from subsampled spiking activity. arXiv preprint arXiv:200703367. 2020;.

[44] Tomen N, Rotermund D, Ernst U. Marginally subcritical dynamics explain enhanced stimulus discriminability under attention. Frontiers in systems neuroscience. 2014;8:151.

[45] Meisel C, Klaus A, Vyazovskiy VV, Plenz D. The interplay between long-and short-range temporal correlations shapes cortex dynamics across vigilance states. Journal of neuroscience. 2017;37(42):10114–10124.

[46] Priesemann V, Valderrama M, Wibral M, Le Van Quyen M. Neuronal avalanches differ from wakefulness to deep sleep–evidence from intracranial depth recordings in humans. PLoS Comput Biol. 2013;9(3):e1002985.

[47] Meisel C. Antiepileptic drugs induce subcritical dynamics in human cortical networks. arXiv:190413026 [q-bio]. 2019;.

[48] Meisel C, Schulze-Bonhage A, Freestone D, Cook MJ, Achermann P, Plenz D. Intrinsic excitability measures track antiepileptic drug action and uncover increasing/decreasing excitability over the wake/sleep cycle. Proceedings of the National Academy of Sciences. 2015;112(47):14694–14699.

[49] Truccolo W, Donoghue JA, Hochberg LR, Eskandar EN, Madsen JR, Anderson WS, et al. Single-neuron dynamics in human focal epilepsy. Nature Neuroscience. 2011;14(5):635–641. doi:10.1038/nn.2782.

[50] Gast H, Niediek J, Schindler K, Boström J, Coenen VA, Beck H, et al. Burst firing of single neurons in the human medial temporal lobe changes before epileptic seizures. Clinical Neurophysiology. 2016;127(10):3329–3334. doi:10.1016/j.clinph.2016.08.010.

[51] Schevon CA, Weiss SA, McKhann Jr G, Goodman RR, Yuste R, Emerson RG, et al. Evidence of an inhibitory restraint of seizure activity in humans. Nature communications. 2012;3:1060.

[52] Falconer M. Mesial temporal (Ammon's horn) sclerosis as a common cause of epilepsy: etiology, treatment, and prevention. The Lancet. 1974;304(7883):767–770.

[53] Badawy R, Macdonell R, Jackson G, Berkovic S. The peri-ictal state: cortical excitability changes within 24 h of a seizure. Brain. 2009;132(4):1013–1021.

[54] Litt B, Lehnertz K. Seizure prediction and the preseizure period. Current opinion in neurology. 2002;15(2):173–177.

[55] Baud MO, Kleen JK, Mirro EA, Andrechak JC, King-Stephens D, Chang EF, et al. Multi-day rhythms modulate seizure risk in epilepsy. Nature communications. 2018;9(1):1–10.

[56] Karoly PJ, Goldenholz DM, Freestone DR, Moss RE, Grayden DB, Theodore WH, et al. Circadian and circaseptan rhythms in human epilepsy: a retrospective cohort study. The Lancet Neurology. 2018;17(11):977–985.

[57] Levina A, Priesemann V. Subsampling scaling. Nature Communications. 2017;8:15140. doi:10.1038/ncomms15140.

[58] Murray JD, Bernacchia A, Freedman DJ, Romo R, Wallis JD, Cai X, et al. A hierarchy of intrinsic timescales across primate cortex. Nature Neuroscience. 2014;17(12):1661–1663. doi:10.1038/nn.3862.

[59] Jirsa VK, Stacey WC, Quilichini PP, Ivanov AI, Bernard C. On the nature of seizure dynamics. Brain. 2014;137(8):2210–2230. doi:10.1093/brain/awu133.

[60] Suffczynski P, Kalitzin S, Da Silva FL. Dynamics of non-convulsive epileptic phenomena modeled by a bistable neuronal network. Neuroscience. 2004;126(2):467–484.

[61] Brinkmann BH, Wagenaar J, Abbot D, Adkins P, Bosshard SC, Chen M, et al. Crowdsourcing reproducible seizure forecasting in human and canine epilepsy. Brain. 2016;139(6):1713–1722.

[62] Cook MJ, O'Brien TJ, Berkovic SF, Murphy M, Morokoff A, Fabinyi G, et al. Prediction of seizure likelihood with a long-term, implanted seizure advisory system in patients with drug-resistant epilepsy: a first-in-man study. The Lancet Neurology. 2013;12(6):563–571.

[63] Freestone DR, Karoly PJ, Cook MJ. A forward-looking review of seizure prediction. Current opinion in neurology. 2017;30(2):167–173.

[64] Niediek J, Boström J, Elger CE, Mormann F. Reliable Analysis of Single-Unit Recordings from the Human Brain under Noisy Conditions: Tracking Neurons over Hours. PLoS ONE. 2016;11(12). doi:10.1371/journal.pone.0166598.

[65] Priesemann V, Levina A, Wilting J. Assessing Criticality in Experiments. In: The Functional Role of Critical Dynamics in Neural Systems. Springer; 2019. p. 199–232.

[66] Jolly E. Pymer4: connecting R and Python for linear mixed modeling. Journal of Open Source Software. 2018;3(31):862.

[67] Bates D, Mächler M, Bolker B, Walker S. Fitting linear mixed-effects models using lme4. arXiv preprint arXiv:14065823. 2014;.
