# Supplementary material for: Assessing criticality in pre-seizure single-neuron activity of human epileptic cortex
Source: PLoS Comput Biol. 2021 Mar 8;17(3):e1008773. doi: 10.1371/journal.pcbi.1008773 (PMC7971851; doi:10.1371/journal.pcbi.1008773)
Supplement: Attachment — Submitted filename: coverletter_replies.pdf [file pcbi.1008773.s013.pdf]

A. Hagemann and V. Priesemann

Max-Planck-Institute for Dynamics and Self-Organization
Am Faßberg 17
D-37077 Göttingen
viola.priesemann@ds.mpg.de

December 22, 2020

Response letter to the reviews of our manuscript

Dear editors,

please find enclosed our newly revised manuscript entitled ”Assessing criticality in pre-seizure single-neuron activity of human epileptic cortex”.
We would like to thank the reviewers for the second feedback and we have addressed the remaining points that were raised. Below you can find detailed list of answers, as well as the implemented changes.

We thank you for your editorial consideration and are looking forward to your reply.

Best regards,

Annika Hagemann, Jens Wilting, Bita Samimizad, Florian Mormann, and Viola Priesemann

Reviewer 1

The authors have responded well to my comments. I just have a few minor suggestions for clarity at this stage, in no particular order:

Please rename "focal" to "ipsi", which also matches the "contra" better and is in agreement with standard epilepsy naming.

We have changed the naming as suggested.

"Our result thus suggests that there can be consistent differences in distance to criticality between hemispheres but that the location of the epileptic focus does not predict that difference." I think you need to be a bit more precise. Either "the location of the epileptic focus does not predict that difference across all patients consistently" and/or "there can be consistent differences in distance to criticality between hemispheres but the direction of the effect was patient-specific".

Thank you for this remark, we now formulate this statement more precisely:

Our result thus suggests that there can be consistent differences in distance to criticality between hemispheres but that the location of the epileptic focus does not predict that difference consistently across patients.

I think the discussion would benefit from a short sentence comment on the fact that within patients, the effect of ipsi vs contra was consistent, and what that would indicate.

Thank you for this input. We now added this aspect to the discussion:

Although there were systematic differences between focal and contra-lateral hemisphere in some patients, these differences were not consistent across patients. Therefore, the observed differences may only reflect patient-specific local differences in the distance to criticality and cannot be attributed to the epileptogenicity of the hemisphere based on our data.

Reviewer 2

I have no objections, and I recommend the manuscript to be accepted for publication in PLOS Computational Biology. The response to the referees was very exciting to read.

Thank you for this positive assessment.

Reviewer 3

The authors have addressed all of my concerns. The manuscript now gives a much more balanced discussion and interpretation of results along with a more comprehensive, balanced overview of the literature. Two minor points to mention (however, I do not need to see the manuscript again):

The discussion of Chang et al., 2018 should not only be limited to the human data results, but should, in all fairness, also mention the animal data results, which displayed clear and strong evidence of critical slowing down (Discussion section, lines 190 and following in the manuscript). Mentioning also these important results would help to provide a more balanced,

also educational overview of the state of evidence to the reader.
We have now added the findings on rat hippocampal slices to the discussion:
*A recent study on ECoG data of human patients with focal epilepsy found clear systematic trends in signatures of critical slowing during the last 30 min before seizure onset, however these trends were not consistent across patients (increase in the signal's autocorrelation coefficient (ACC) in 4/12 patients, decrease in 4/12 and no significant change in the ACC in 4/12 patients) [**?**]. High frequency activity in hippocampal slices of rats, on the other hand, showed a consistent increase in the ACC as well as other signatures of critical slowing prior to seizures [**?**].*

Reference [47] has meanwhile been published and should be cited accordingly: [47] Antiepileptic drugs induce subcritical dynamics in human cortical networks. Christian Meisel. Proceedings of the National Academy of Sciences May 2020, 117 (20) 11118-11125;
DOI: 10.1073/pnas.1911461117
Thank you for this remark, we have corrected the reference as requested.

Reviewer 4
The authors have addressed my concerns.
Thank you for this positive assessment.
